# Autonomous emergency braking optimal coordination control strategy for commercial buses considering safety and comfort

**DOI:** 10.1371/journal.pone.0357272

**Published:** 2026-09-11

**Authors:** Shoulin Gao, Xingyu Liu, Jianlong Hu, Chaohui Yang, Zewen Yang, Kai Jiang

**Affiliations:** 1 School of Transportation and Vehicle Engineering, Shandong University of Technology, Zibo, China; 2 Tsingte Group Co., Ltd, Qingdao, China; National University of Singapore, SINGAPORE

## Abstract

The traditional dual-stage warning and dual-grade braking autonomous emergency braking (AEB) control strategy has sudden change in the expected braking deceleration during the switch of working state, which will easily lead to significant longitudinal jerk of the vehicle and affect ride comfort during emergency braking. To solve this problem, an AEB optimal control strategy considering coordination safety and comfort is proposed. First, an in improved Time-To-Collision pre-estimation model was designed, and the AEB state threshold time determination method was proposed by combining vehicle braking process and driver emergency braking characteristics. Second, an AEB hierarchical control strategy was designed, the upper layer designed an expected braking deceleration optimal decision controller based on particle swarm optimization linear quadratic regulator (PSO-LQR), and introduced a comfort time compensation coefficient to rectification the PSO-LQR controller. Moreover, an expected braking deceleration tracking controller based on feedforward and robust integral sliding mode feedback control was designed in the lower layer. Next, an AEB software architecture based on AUTOSAR was designed to enhance the standardization of control strategy software code generation. Finally, the Hardware-in-the-loop test and real vehicle experimental results indicate that the proposed AEB optimal coordination control strategy can meet the safety requirements of emergency collision avoidance functions, and effectively reduce the longitudinal jerk compared to the traditional AEB control strategy, it significantly improves the ride comfort of commercial buses during emergency braking processes.

## 1. Introduction

At present, the active safety control technology of automobiles has received increasing attention [1,2]. According to statistics, the frequency of casualties in the traffic system, especially pedestrians or elderly vulnerable groups, is relatively high [3,4]. With the development of automobile technology, automatic driving has become an effective way to solve traffic accidents [5]. Autonomous emergency braking (AEB) system is an intelligent control technology for autonomous vehicles, which can effectively preventing collision related accidents and enhancing the active safety of vehicles [6].

Nowadays with the gradual improvement of vehicle intelligence level, it has become a trend for commercial buses to be equipped with AEB. For example: the European Union (EU) General Safety Regulation require that from January 2022, commercial and tourist buses with vehicle lengths of more than 11 meters must to be installed AEB system [7]; the National Highway Traffic Safety Administration (NHTSA) of the United States also plans to make AEB a standard configuration for all new passenger cars and light trucks by 2029 [8].

The core principle of AEB control technology is to judge or predict the collision risk of vehicles in real time, and then make reasonable emergency braking action according to the predicted collision risk level. Therefore, accurately judging the vehicle's collision risk and determine the reasonable expected braking deceleration are the keys to AEB. At present, the AEB control strategies are mainly divided into two categories based on collision risk prediction methods, one is the safety distance model, and the other is the time-to-collision (TTC) model.

The safety distance model is that the AEB system using the relative distance between the ego vehicle and the lead vehicle or obstacle as the basis for triggering warning and emergency braking states [9,10]. The safety distance model refers to the AEB system using the relative distance between the ego vehicle and the lead vehicle or obstacle as the basis for triggering warning and emergency braking states. The classic safety distance models mainly include the Honda model [11], Mazda model [12], SeungwukMoon model [13], and the Berkeley model proposed by the University of Berkeley [14]. Many scholars have conducted research on AEB control strategies based on the safety distance model. Ko et al. [15] proposed an AEB control strategy that considers vehicle mass estimation to improve the accuracy of braking distance prediction, which effectively improves the correctness of the AEB working state determination. In reference [16], a multi-stage warning and autonomous braking control strategy based on collision safety distance as the evaluation index is proposed, and a safety distance curve compensation coefficient is designed, which effectively improving the active collision avoidance performance under turning conditions. However, the AEB control strategy based on the safety distance model only considers the inter-vehicle relative motion state, ignoring the driver characteristics and the ride comfort during the braking process. To solve this problem, Li et al. [17] proposed a self-supervised contrastive learning method for driving style recognition, designed an AEB control strategy that considers driver personality, and compared with classic safety distance model control strategies such as Mazda and Honda. The results showed that the proposed control strategy could improve the comfort during braking. Seungho et al. [18] proposed a self-adaptive safety distance model, which adjusts the safety distance utilize the uncertainty of the braking duration of the target vehicle, thereby improving driving comfort.

The safety distance model only considers the factor of distance, which has poor applicability in complex road conditions and will affect the driving experience. Compared to the safety distance model, the TTC model adopts pre-estimated collision time as the basis for determining the AEB working state, which can better reflect the driver's perception and experience of collision risk. In reference [19], a priority-based framework is developed to identify high-risk locations across space and time using the space–time cube method, which provided practical guidance for transportation engineers and safety planners to enhance safety outcomes for vulnerable road users. Lan et al. [20] proposed a TTC graded braking deceleration threshold that considers braking comfort by analyzing the emergency braking data of the driver. However, the graded braking strategy cannot completely avoid the problem of significant longitudinal jerk caused by sudden changes in expected braking deceleration. In reference [21], a TTC graded braking deceleration control strategy based on road adhesion coefficient recognition and vehicle mass estimation is designed, which improves the adaptability of AEB under different working conditions. However, the above references mentioned AEB graded braking control strategy based on TTC model has a sudden change in expected braking deceleration during the process of braking state changes, which would affect the ride comfort. To improve the comfort during emergency braking, Liu et al. [22] proposed an AEB adaptive layered control strategy based on fuzzy rule model prediction algorithm. However, the fuzzy control lacks strict mathematical theoretical basis, and the design of membership functions excessive reliance on expert experience. In reference [23], an AEB control strategy for semi-trailer trains based on BP neural network to predict collision time TTC, which improves the safety and comfort of emergency braking of vehicles. Bae et al. [24] proposed an AEB control strategy that considers both safety and ride comfort based on the TTC model. It achieves safe deceleration through rapid control of brake pressure, and utilizes smooth release of brake pressure to achieve great ride comfort, but it cannot effectively improve the braking comfort of the entire emergency braking process.

Summarizing the above research, there are two problems in the existing AEB graded braking control strategy based on safety distance model and TTC model. One problem is that the traditional TTC model only considers relative distance and relative speed factors, which causes the calculated TTC to tend to infinity when the speed of the ego vehicle and lead vehicle are very close. Moreover, the acceleration relationship between ego vehicle and lead vehicle is not considered, making it impossible to accurately judge the collision risk level. Another problem is that the expected braking deceleration of the existing AEB control strategy would have discontinuous change in the process of emergency braking state switching, which can easily lead to significant longitudinal jerk and affect the passenger ride comfort during emergency braking. Commercial buses have higher requirements for safety and driving comfort during emergency braking due to their unique large-scale passenger transport function. To solve the above problems, this paper focus on researching the AEB control strategy that considers dynamic coordination between collision avoidance safety and braking comfort by making the following contributions or innovations.

1) An improved TTC model is proposed and an AEB state threshold time calculation method is designed that combines braking process analysis and driver emergency braking characteristic statistics, which provides a foundation for accurate decision-making on AEB control system working states.
2) An AEB optimal coordination control strategy considering safety and comfort based on inter-vehicle motion model is proposed, with an expected braking deceleration optimal decision controller based on PSO-LQR and comfort time compensation coefficient established in the upper layer, and an expected braking deceleration robust tracking controller is designed in the lower layer, which improved the adaptability and robustness during emergency braking control of vehicles.

The remaining sections of this paper are organized as follows. The vehicle and braking system models are established in Section 2. In Section 3 designs AEB control scheme and improved TTC model. The AEB hierarchical control strategy is established in Section 4. The HIL test and real vehicle experiment validation of the proposed control strategy is carried out in Section 5. Finally, the conclusions drawn can be found in Section 6.

## 2. Vehicle and brake system modeling

In this section, a vehicle longitudinal dynamic model during braking process and a commercial buses braking system model are established, which provides a foundational platform for the design and verification of AEB control strategy.

### 2.1. Vehicle longitudinal dynamics modeling

In the process of emergency braking, the vehicle involves only the braking deceleration control in the longitudinal motion direction, and the vehicle dynamic equation can be expressed as [25]:


$$\begin{array}{c} \left\{ \begin{array}{c} @l@F_{b}=F_{i}+F_{w}+F_{f}+F_{j}\\ F_{b}=T_{b}/r\\ F_{i}=mg\;sin\beta \\ F_{w}=C_{D}A\rho v^{\text{2}}/2\\ F_{j}=m\dot{v}=ma_{b} \end{array}\right. \end{array}$$ (1)

where, $F_{b}$, $F_{i}$, $F_{w}$, $F_{f}$ and $F_{j}$ are the vehicle braking force, gradient resistance, air resistance, rolling resistance and acceleration resistance, respectively; $T_{b}$ denotes the braking torque; r is the tire rolling radius; m represents the vehicle mass; β is the road gradient; $C_{D}$ and ρ are the air drag coefficient and air density, respectively; A is the front area; v is the vehicle speed; $a_{b}$ represents the vehicle deceleration.

### 2.2. Pneumatic braking system modeling

**Fig 1:**
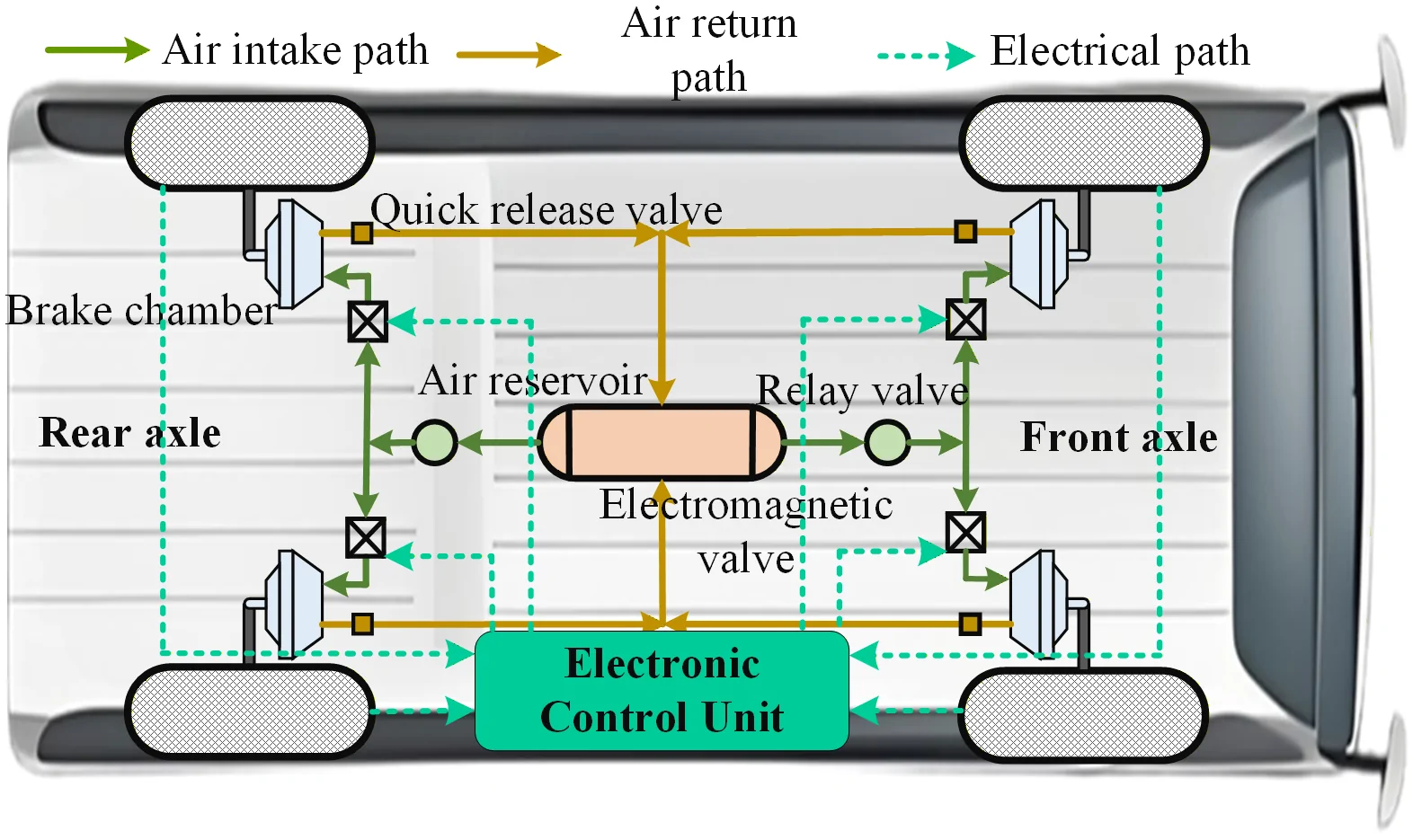
Schematic diagram of pneumatic braking system.

AEB controls the braking system pressure to achieve the vehicle’s excepted braking deceleration. The commercial buses studied in this paper adopts pneumatic braking system [26], which as shown in the Fig 1. The braking system is mainly composed of air reserve, relay valve, electronic magnetic valve, brake chamber, quick release valve and Electronic Control Unit.

(1) Air reservoir model

Air reservoir as an energy storage component in pneumatic braking system, the gas pressure would decrease with the increase of the pressure of each component in the pneumatic circuit, so it is necessary to consider the pressure change of the air reservoir during vehicle braking. According to the thermodynamic theory of variable mass system and the first law of thermodynamics, the energy change in the air reservoir can be obtained as follows:


$$\begin{array}{c} dU=c_{v}T_{a}(q_{in}-q_{out})+m_{a}c_{v}\frac{dT_{a}}{dt} \end{array}$$ (2)

where, $c_{v}$ is the specific heat capacity at constant volume; $T_{a}$ is the gas temperature; $m_{a}$ represents the gas mass; $q_{in}$ and $q_{out}$ are the flow of gas into and out of the air reservoir.

According to the law of thermodynamic, the gas temperature $T_{a}$ can be obtained as follows:


$$\begin{array}{c} \left\{ \begin{array}{c} @l@\frac{dT_{a}}{dt}=\frac{R_{a}T_{a}}{p_{\text{0}}V_{a}}q_{in}\left(\gamma_{a}T_{q}-T_{a}\right)-\frac{R_{a}T_{a}^{\text{2}}}{p_{\text{0}}V_{a}}q_{out}(\gamma_{a}-1)\\ \text{\textbackslash{}hspace\{0.33em}\;\;\;\}-(\gamma_{a}-1)\frac{T_{a}}{V_{a}}\frac{dV_{a}}{dt}+(\gamma_{a}-1)\frac{T_{a}\dot{Q}}{p_{\text{0}}V_{a}}\\ R_{a}=c_{p}-c_{v}\\ \gamma_{a}=c_{p}/c_{v} \end{array}\right. \end{array}$$ (3)

where, $p_{a}$ is the air reservoir gas pressure; $V_{a}$ denotes the air reservoir volume; Q is the heat exchange between the air reservoir and the outside; $c_{p}$ is the specific heat capacity at constant pressure; $T_{q}$ denotes the temperature of the gas flowing out of the air reservoir, which can be expressed as:


$$\begin{array}{c} T_{q}=T_{ip}{\left(\frac{p_{\text{0}}}{p_{ip}}\right)}^{\left(c_{p}/c_{v}-1\right)/\left(c_{p}/c_{v}\right)} \end{array}$$ (4)

where, $p_{\text{0}}$ is the atmospheric absolute pressure; $T_{iq}$ and $p_{ip}$ are the temperature and pressure of the air reservoir gas.

According to the time differential equation of the state equation, the change of gas pressure in the air reservoir can be calculated as:


$$\begin{array}{c} \frac{dp_{a}}{dt}=\frac{R_{a}}{V_{a}}q_{in}T_{q}-\frac{R_{a}T_{a}}{V_{a}}q_{out}\gamma_{a}-(\gamma_{a}-1)\frac{p_{a}}{V_{a}}\frac{dV_{a}}{dt}+(\gamma_{a}-1)\frac{T_{a}\dot{Q}}{p_{a}V_{a}} \end{array}$$ (5)

(2) Relay valve modeling

In the pneumatic braking system, the main function of relay valve is to shorten the air intake distance, and the motion equation of relay valve can be expressed as:


$$\begin{array}{c} \left\{ \begin{array}{c} @l@m_{\text{1}}{\ddot{x}}_{\text{1}}=A_{\text{1}}p_{\text{1}}-A_{\text{2}}p_{\text{2}}\underset{―}{+}C_{\text{1}}{\dot{x}}_{\text{1}}-m_{\text{1}}g\text{\textbackslash{}hspace\{1em}\;\;\;\;\;\}(0\leq x_{\text{1}}\leq x_{\text{0}})\\ (m_{\text{1}}+m_{\text{2}}){\ddot{x}}_{\text{1}}=A_{\text{1}}p_{\text{1}}-A_{\text{2}}p_{\text{2}}\underset{―}{+}(C_{\text{1}}+C_{\text{2}}){\dot{x}}_{\text{1}}-F_{\text{0}}\\ \text{\textbackslash{}hspace\{1em}\;\;\;\;\;\}-K_{\text{1}}(x_{\text{1}}-x_{\text{0}})-(m_{\text{1}}+m_{\text{2}})g\text{\textbackslash{}hspace\{0.5em}\;\}(x_{\text{0}}\leq x_{\text{1}}\leq x_{max}) \end{array}\right. \end{array}$$ (6)

where, $m_{\text{1}}$ and $m_{\text{2}}$ are the mass of the piston and valve core, respectively; $x_{\text{1}}$ denotes the displacement of the piston; $p_{\text{1}}$ and $p_{\text{2}}$ represent the air pressure in the upper and lower chambers of the piston; $A_{\text{1}}$ and $A_{\text{2}}$ are the effective areas of the upper and lower chambers of the piston, respectively; $C_{\text{1}}$ and $C_{\text{2}}$ are the viscous drag coefficients of the upper and lower chambers of the piston; $x_{\text{0}}$ represents the gap between the piston and the valve core; $F_{\text{0}}$ is the valve core spring preload.

(3) Electromagnetic valve modeling

The electromagnetic valve adjusts the pressure of the brake chamber by controlling the gas flow rate, and the gas flow equation of the electromagnetic valve can be expressed as:


$$\begin{array}{c} {\dot{q}}_{e}=k_{e}C_{e}A_{e}\sqrt{\frac{2{\dot{p}}_{e}}{\rho_{e}}} \end{array}$$ (7)

where, $k_{e}$ is the duty cycle; $C_{e}$ represents the gas flow coefficient; $A_{e}$ denotes the valve hole area; $\rho_{e}$ and $P_{e}$ are the gas density and pressure, respectively.

(4) brake chamber

The wheel braking force is controlled by adjusting brake chamber pressure. The commercial buses studied in this paper uses diaphragm spring brake chamber on both the front and rear axles, and the pressure change i during the braking process can be formulated as [27]:


$$\begin{array}{c} {\dot{p}}_{bc}=\left\{ \begin{array}{c} @l@\frac{k_{\text{0}}A_{b}p_{bup}}{V_{b}}{\left(\frac{\text{2}}{k_{\text{0}}+1}\right)}^{\frac{\text{1}}{k_{\text{0}}-1}}\sqrt{\frac{2k_{\text{0}}R_{\text{0}}T_{\text{0}}}{k_{\text{0}}+1}}\text{\textbackslash{}hspace\{1em}\;\;\;\;\;\}\frac{p_{bdown}}{p_{bup}}<\delta_{\text{0}}\\ \frac{k_{\text{0}}A_{b}p_{bup}}{V_{b}}\sqrt{\frac{2k_{\text{0}}R_{\text{0}}T_{\text{0}}}{k_{\text{0}}-1}{\left(\frac{p_{bdown}}{p_{bup}}\right)}^{\frac{\text{2}}{k_{\text{0}}}}-{\left(\frac{p_{bdown}}{p_{bup}}\right)}^{\frac{k_{\text{0}}+1}{k_{\text{0}}}}}\text{\textbackslash{}hspace\{1em}\}\frac{p_{bdown}}{p_{bup}}\geq \delta_{\text{0}}\text{\textbackslash{}hspace\{1em}\} \end{array}\right. \end{array}$$ (8)

where, $k_{\text{0}}$ represents the adiabatic coefficient, $k_{\text{0}}=0.4$; $R_{\text{0}}$ is the gas constant, $R_{\text{0}}=284J/kg$; $A_{\text{0}}$ denotes the flow area; $T_{\text{0}}$ is the gas absolute temperature; $V_{b}$ represents the brake chamber volume; $p_{bdown}$ and $p_{bup}$ are the upstream and downstream pressure of the brake chamber, respectively; $\delta_{\text{0}}$ is the critical pressure ratio, $\delta_{\text{0}}=0.52$.

(5) Quick release valve

Quick release valve can accelerate the deflation process when the braking is released, and its motion equation can be expressed as:


$$\begin{array}{c} m_{q}{\ddot{x}}_{q}=p_{qin}A_{qin}-p_{qout}A_{qout}-F_{q} \end{array}$$ (9)

where, $x_{q}$ and $m_{q}$ are the diaphragm displacement and mass, respectively; $p_{qin}$ and $p_{qout}$ are the gas intake and outlet chamber pressure, respectively; $A_{qin}$ and $A_{qout}$ are the gas intake and outlet chamber area, respectively; $F_{q}$ represents the diaphragm deformation force.

**Fig 2:**
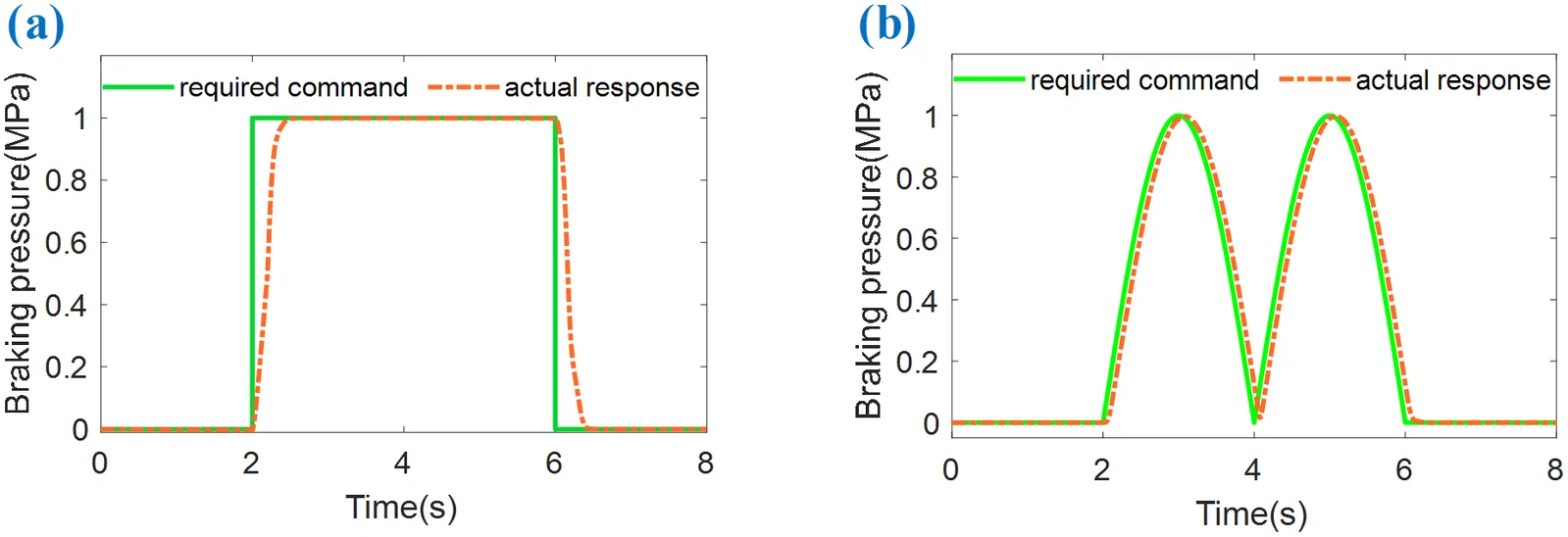
Dynamic response performance of pneumatic braking system. (a) step input. (b) sinusoidal input.

Under the two typical braking pressure required conditions of step and sinusoidal input command, the dynamic response performance of the established pneumatic braking system model is analyzed, and the simulation results are shown in Fig 2. Under two typical operating conditions, the response time of the pressure increase and decrease process of the brake chamber is within 0.5 seconds, and the response speed of the brake pressure can meet the requirements of the AEB control system.

## 3. AEB control scheme and state threshold time determination

In this section, an AEB control scheme of dual-stage warning and single-grade braking (DW-SB) that considers braking comfort is proposed, an improved time-to-collision (TTC) calculation model considering inter-vehicle relative deceleration is designed, and analyze the process of determining the AEB state threshold time, which provides an overall framework for the design of AEB control strategy.

### 3.1. AEB control scheme

**Fig 3:**
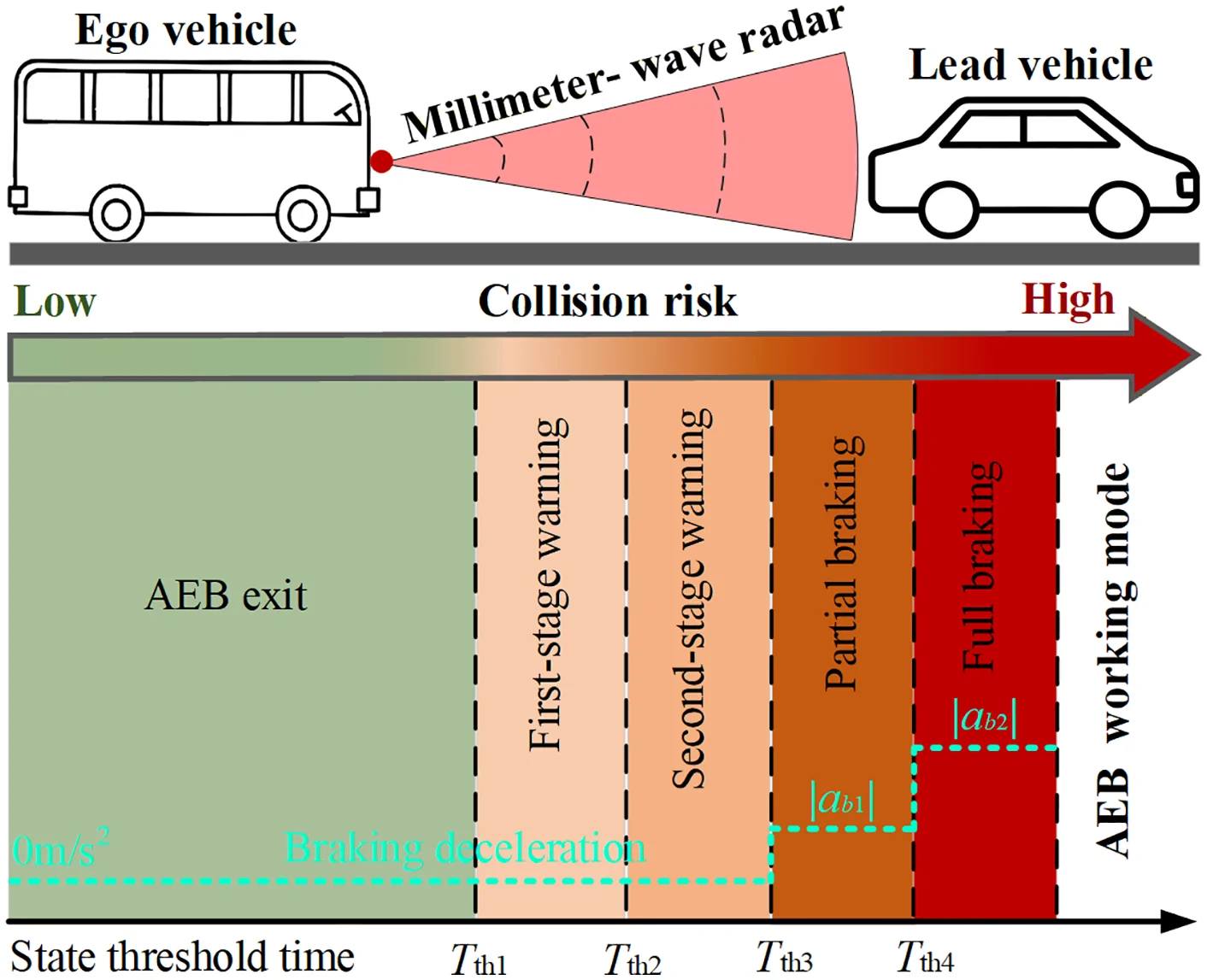
The traditional DW-DB AEB control structure.

At present, AEB generally adopts a dual-stage warning and dual-grade braking (DW-DB) control scheme [23], [28], [29], and its control structure is shown in Fig 3. The control principle of DW-DB is to first use millimeter wave radar and other related sensors to collect the driving information of the ego vehicle and lead vehicle, and real time calculating the pre-estimated TTC $t_{TTC}$ between the ego vehicle and the obstacles ahead. Next, the $t_{TTC}$ is compared with the AEB state threshold time $T_{thi}\text{\textbackslash{}hspace\{0.17em}\}(i=1,2,3,4)$ to determine the vehicle's collision risk level.

From the Fig 3, it can be seen that the traditional DW-DB AEB control scheme determines a fixed expected braking deceleration based on the vehicle collision risk level, which has the advantage of a simple control method and easy to applied in engineering. However, when the DW-GB AEB control strategy enters partial braking from the warning state, or enters full braking from the partial braking state. The expected braking deceleration determined by the AEB control strategy has a sudden change from 0 to $a_{b1}$ or $a_{b1}$ to $a_{b2}$, which would result in a large longitudinal jerk on the vehicle, and ultimately affecting the passenger comfort during the emergency braking process.

**Fig 4:**
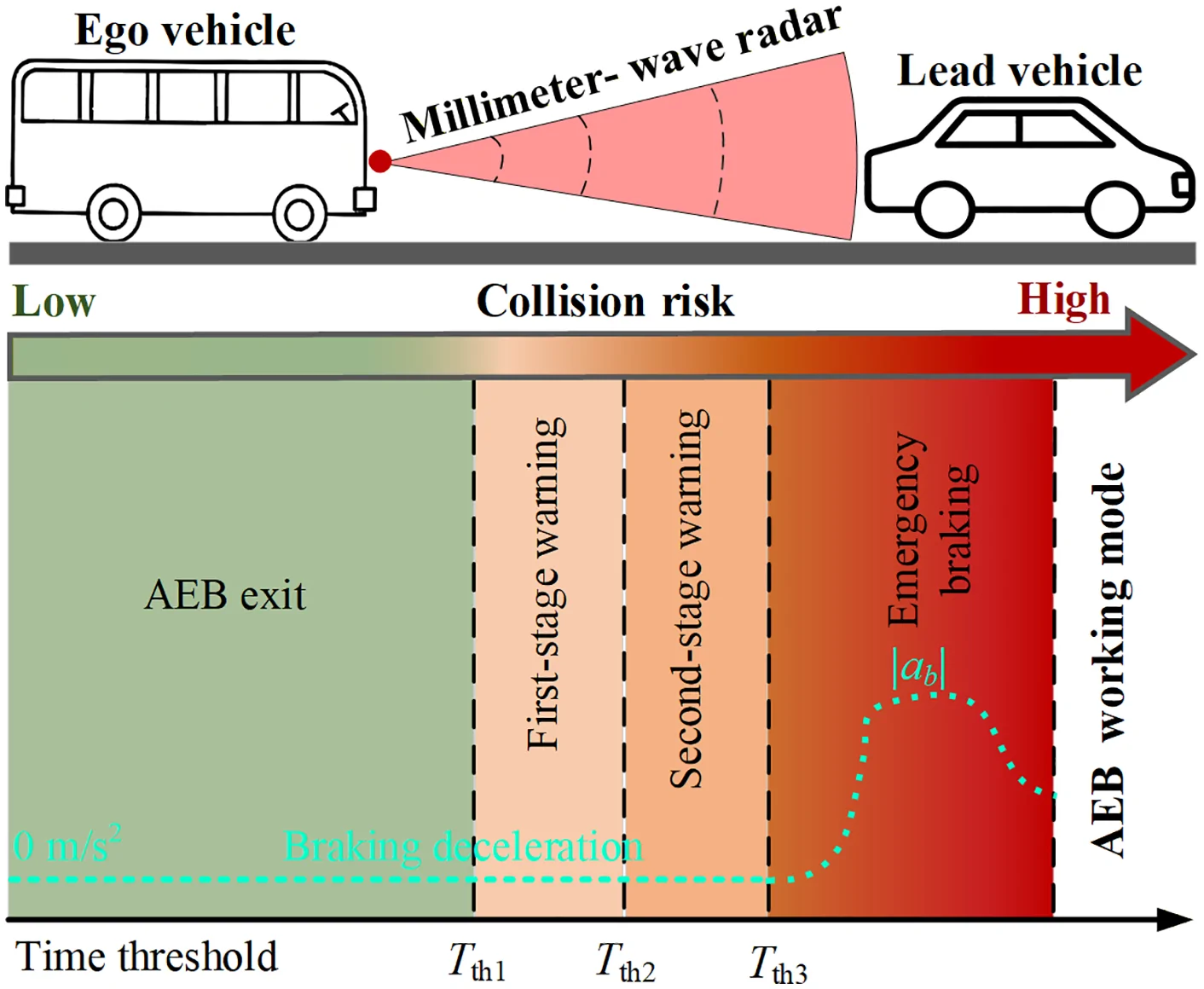
The proposed DW-SB AEB control scheme.

To solve this question, a DW-SB AEB control scheme has been proposed, and its control structure is shown in Fig 4. According to the collision risk of the vehicle and passenger's riding comfort, the DW-SB control scheme can adaptively adjust the vehicle’s expected braking deceleration $a_{b}$ during emergency braking. It can achieve adaptive smooth changes in $a_{b}$ according to the collision risk of the vehicle, so as to reduce the sudden changes in expected braking deceleration during state changes in traditional DW-DB AEB control scheme. Therefore, while ensuring the safety of bus collision avoidance, it can significantly improve the driving comfort of the vehicle.

### 3.2. Improved TTC model

Real time acquisition of pre-estimated TTC is an important basis for AEB system. TTC is defined as the time it takes for the ego vehicle and the lead vehicle to maintain their current driving state and continue driving until a collision occurs [30]. In the process of driving, the relative speed between the ego vehicle and the lead vehicle can be expressed as:


$$\begin{array}{c} v_{r}=v_{l}-v_{e} \end{array}$$ (10)

where, $v_{e}$ represents the ego vehicle speed, $v_{l}$ is the lead vehicle speed.

When the speed of the ego vehicle is greater than that of the lead vehicle, the vehicles would collide if there is no braking deceleration. At this time, the TTC can be calculated as:


$$\begin{array}{c} t_{TTC}=\frac{d_{r}}{v_{r}} \end{array}$$ (11)

where, $d_{r}$ denotes the inter-vehicle relative distance.

Utilization Equation (11) calculates the TTC only considers the two factors of inter-vehicle relative distance and relative speed. When the speed between the ego vehicle and lead vehicle are very close, that is, the $v_{r}$ is closed to zero, the calculated TTC would tend to be infinite. Moreover, duo to the inter-vehicle relative acceleration or deceleration relationship is ignored, it is impossible to accurately estimate the collision risk level of the vehicles.

Therefore, taking into account the deceleration between the ego vehicle and lead vehicle, an improved second-order TTC calculation method based on inter-vehicle relative deceleration is designed. First, the relative motion process of the vehicles is simplified, that is, the ego vehicle and lead vehicle maintain their current vehicle speed and acceleration until a collision occurs. At this time, the inter-vehicle relative deceleration can be expressed as:


$$\begin{array}{c} a_{r}=a_{e}-a_{l} \end{array}$$ (12)

where, $a_{e}$ and $a_{l}$ represents the braking deceleration of ego vehicle and lead vehicle, respectively. $a_{e}$ is obtained in real time by its oneself inertial measurement unit (IMU). Millimeter wave radar transmits millimeter wave signals and receives the reflected signals to determine the distance and speed of the target object, the $a_{l}$ is obtained by differentiating the speed information of the lead vehicle obtained by millimeter wave radar.

When a collision occurs, the relationship between the ego vehicle driving distance $d_{e}$, the lead vehicle driving distance $d_{l}$, and the inter-vehicle relative distance $d_{r}$ is as follows:


$$\begin{array}{c} \left\{ \begin{array}{c} @l@d_{e}=d_{l}+d_{r}\\ d_{e}=a_{e}t^{\text{2}}/2+v_{e}t\\ d_{l}=a_{l}t^{\text{2}}/2+v_{l}t \end{array}\right. \end{array}$$ (13)

By substituting Equations (10) and (12) into Equation (13), it can be obtained as follows:


$$\begin{array}{c} a_{r}t^{\text{2}}/2+v_{r}t-d_{r}=0 \end{array}$$ (14)

The positive real number solution of Equation (14) is TTC, which can be formulated as follows:


$$\begin{array}{c} t_{TTC}=\left\{ \begin{array}{c} @l@\frac{d_{r}}{v_{r}},\;\;\;\;\;\;\;v_{r}>0,\;\;a_{r}=0\\ \frac{-v_{r}-\sqrt{v_{r}^{\text{2}}-2a_{r}d_{r}}}{a_{r}},\;\;\;v_{r}<0,\;\;\;a_{r}\neq 0\\ \frac{-v_{r}+\sqrt{v_{r}^{\text{2}}-2a_{r}d_{r}}}{a_{r}},\;\;\;v_{r}\geq 0,\;\;a_{r}<0\\ t_{c}\;\;\;\;\;\;\;\;else \end{array}\right. \end{array}$$ (15)

where, $t_{c}$ is the constant, which is usually $t_{c}\geq T_{th1}$.

It should be pointed out that it is similar to the traditional TTC model, the correct calculation of the improved TTC model depends on the accuracy of the information collection of the in-vehicle sensor. When the signal of the on-board sensor is distorted, the calculation of the improved TTC model may be inaccurate.

### 3.3. AEB state threshold time determination

(1) Vehicle braking process analysis

**Fig 5:**
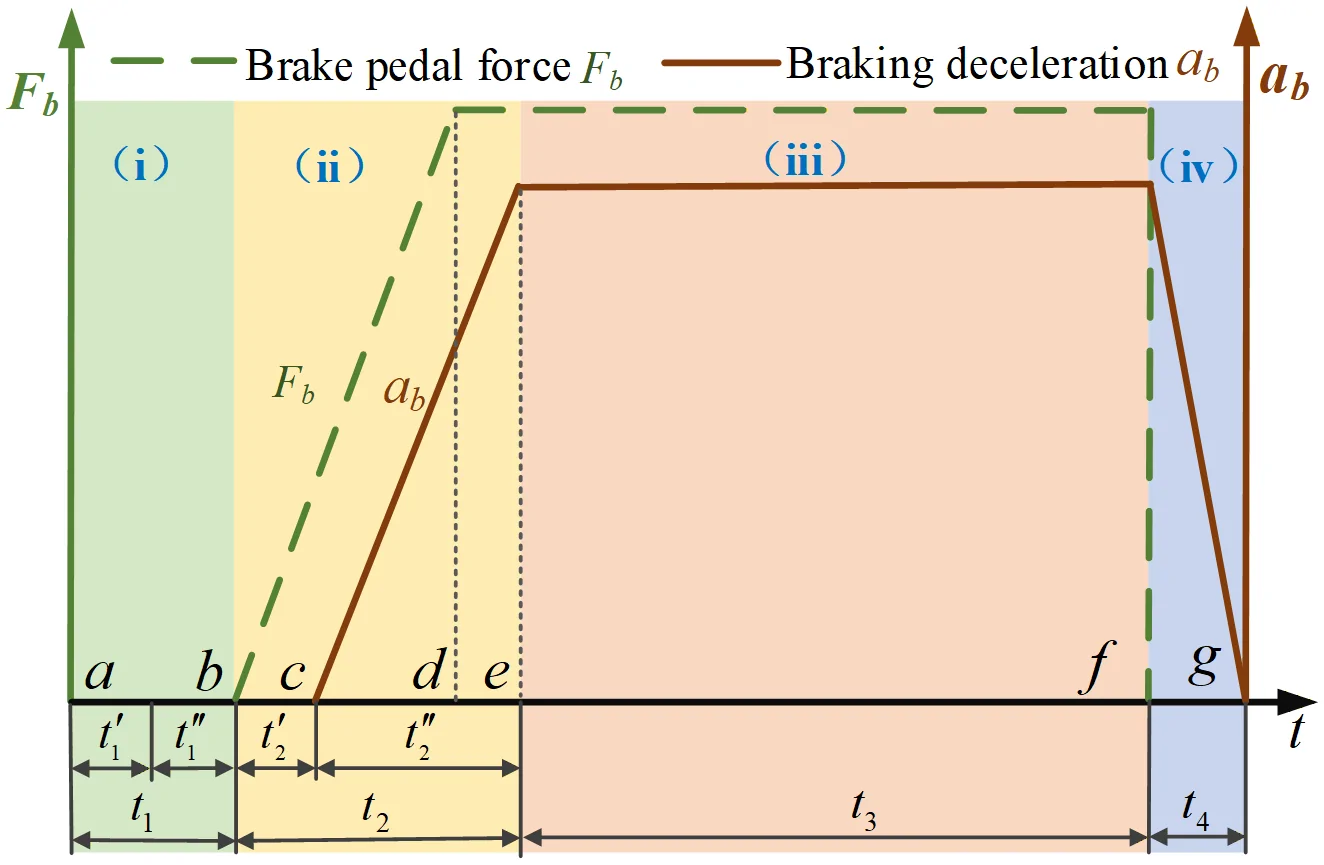
The braking process of vehicles.

When the driver finds an abnormal situation ahead, the process from the start of deceleration to the vehicle being stationary is shown in Fig 5. The entire braking process is mainly divided into four stages: driver response, braking action, continuous braking and braking force release [31].

i) Driver response stage: it represents the time from the driver becoming aware of a dangerous situation to starting to press the brake pedal, which as shown from point *a* to point *b* in Fig 5. At this stage, the vehicle still maintains a constant speed with an initial speed of $v_{\text{0}}$, and the distance traveled by the vehicle can be formulated as:

$$\begin{array}{c} \left\{ \begin{array}{c} @l@d_{\text{1}}=v_{\text{0}}t_{\text{1}}\\ t_{\text{1}}={t^{\prime }}_{\text{1}}+{t^{\prime \prime }}_{\text{1}} \end{array}\right. \end{array}$$ (16)

where, $t_{\text{1}}$ represents the driver response time, which ranges is from 0.3s to 1.0s; ${t^{\prime }}_{\text{1}}$ denotes the brain response time; ${t^{\prime \prime }}_{\text{1}}$ is the response time of stepping on the brake pedal.

ii) Braking action stage: it represents the time from the driver stepping on the brake pedal to generate the continuous braking time, which as shown from point *b* to point *d* in Fig 5.

As displayed from point *b* to point *c* in Fig 5, it indicates that the time for the braking system to overcome the braking gap is ${t^{\prime }}_{\text{2}}$. During this time, the vehicle maintains a constant speed of $v_{\text{0}}$ without braking deceleration, and the travels distance can be expressed as:


$$\begin{array}{c} {d^{\prime }}_{\text{2}}=v_{\text{0}}{t^{\prime }}_{\text{2}} \end{array}$$ (17)

As depicted from point *c* to point *d* in Fig 5, the braking system begins to generate braking pressure until the target pressure is reached, the distance traveled by the vehicle can be formulated as:


$$\begin{array}{c} {d^{\prime \prime }}_{\text{2}}=\int_{\text{0}}^{{t^{\prime \prime }}_{\text{2}}}(v_{\text{0}}-\frac{a_{b}}{2{t^{\prime \prime }}_{\text{2}}}t^{\text{2}})dt=v_{\text{0}}{t^{\prime \prime }}_{\text{2}}-\frac{a_{b}}{\text{6}}{t^{\prime \prime }}_{\text{2}}^{\text{2}} \end{array}$$ (18)

where, $a_{b}$ represents the target braking deceleration; ${t^{\prime \prime }}_{\text{2}}$ is the time from generating braking force to reaching the target braking deceleration.

Therefore, during the entire brake action stage, the vehicle's travel distance can be reorganized as:


$$\begin{array}{c} d_{\text{2}}={d^{\prime }}_{\text{2}}+{d^{\prime \prime }}_{\text{2}}=v_{\text{0}}{t^{\prime }}_{\text{2}}+v_{\text{0}}{t^{\prime \prime }}_{\text{2}}-\frac{a_{b}}{\text{6}}{t^{\prime \prime }}_{\text{2}}^{\text{2}} \end{array}$$ (19)

iii) Continuous braking stage: the pressure of the braking system reaches the target pressure and remains constant, the braking deceleration remains constant $a_{b}$ until the driver releases the brake pedal, which as illustrated from point e to point f in Fig 5. At that time, the vehicle's travel distance can be expressed as:

$$\begin{array}{c} d_{\text{3}}=\frac{v_{\text{0}}^{\text{2}}}{2a_{b}}-\frac{v_{\text{0}}{t^{\prime \prime }}_{\text{2}}}{\text{2}}+\frac{a_{b}{t^{\prime \prime }}_{\text{2}}^{\text{2}}}{\text{8}} \end{array}$$ (20)

iv) braking force release stage: after the driver releases the brake pedal, it takes some time for the braking force to dissipate. At this stage, the deceleration rapidly decreases from $a_{b}$ to 0, the vehicle speed is very small and approaching zero. So, the driving distance of vehicles can be ignored, that is, $S_{\text{4}}\approx 0$.

In summary, through a detailed analysis of the vehicle braking process, the braking distance can be expressed as:


$$\begin{array}{c} \begin{array}{c} d_{t}=d_{\text{1}}+d_{\text{2}}+d_{\text{3}}+d_{\text{4}}\\ \text{\textbackslash{}hspace\{0.17em}\;\;\;\}=v_{\text{0}}t_{\text{1}}+v_{\text{0}}{t^{\prime }}_{\text{2}}+\frac{v_{\text{0}}}{\text{2}}{t^{\prime \prime }}_{\text{2}}-\frac{a_{b}}{24}{t^{\prime \prime }}_{\text{2}}^{\text{2}}+\frac{v_{\text{0}}^{\text{2}}}{2a_{b}} \end{array} \end{array}$$ (21)

Since AEB does not need to consider the driver's response time $t_{\text{1}}$ and ${t^{\prime \prime }}_{\text{2}}$ is usually very small, $t_{\text{1}}$ and ${t^{\prime \prime }}_{\text{2}}^{\text{2}}$ in Equation (21) can be ignored, and the braking distance of the vehicle can be simplified as:


$$\begin{array}{c} d_{t}=v_{\text{0}}{t^{\prime }}_{\text{2}}+\frac{v_{\text{0}}}{\text{2}}{t^{\prime \prime }}_{\text{2}}+\frac{v_{\text{0}}^{\text{2}}}{2a_{b}} \end{array}$$ (22)

(2) State threshold time calculation

**Fig 6:**
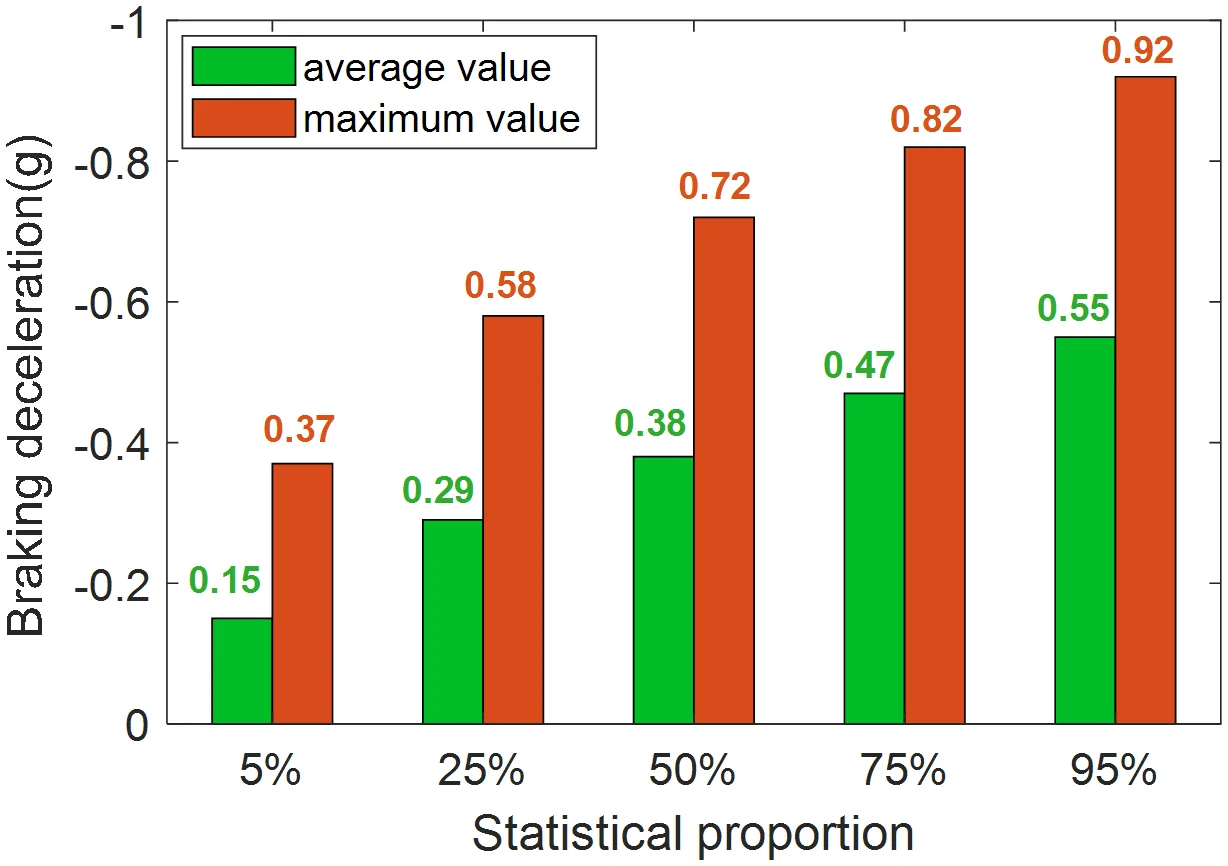
NHTSA statistical results of the driver emergency braking deceleration.

As shown in Fig 3 and Fig 4, the braking deceleration and the state threshold time are key parameters of AEB control scheme. The intensity of braking deceleration has a significant impact on the safety and comfort during braking. The NHTSA conducted data statistics on the braking deceleration of drivers during braking [32], the results as shown in Fig 6.

As described in Fig 6, we can find that 50% of drivers apply an average braking deceleration of less than −0.38g during emergency braking, and 95% of drivers experience a maximum braking deceleration of less than −0.92g. Based on the statistical results of NHTSA, refer to the average braking deceleration of most drivers during emergency braking, while considering the maximum braking deceleration constraint for comfort. The braking deceleration for partial braking and full force braking in the traditional DW-DB control scheme (as shown in Fig 3) is $a_{b1}=-0.4g$ and $a_{b2}=-0.6g$, respectively.

**Fig 7:**
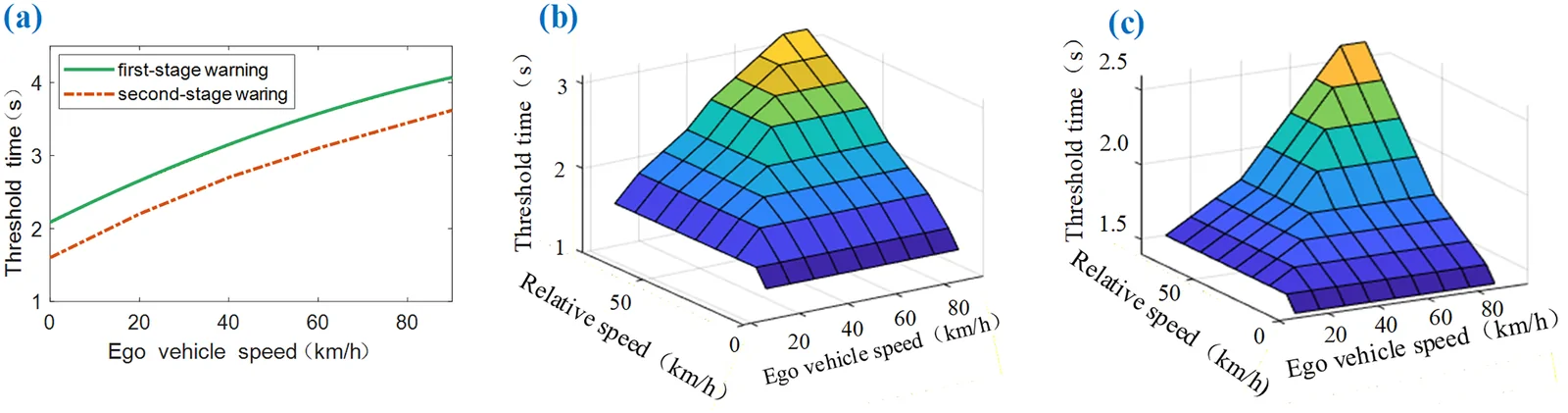
AEB control scheme states threshold time. (a) Warning state threshold time $T_{th1}$ and $T_{th2}$. (b) Partial braking state threshold time $T_{th3}$. (c) Full braking state threshold time $T_{th4}$.

Due to the vehicle does not carry out emergency braking operation in the warning state, the determination method of the first-stage warning threshold time $T_{th1}$ and the second-stage warning threshold time $T_{th2}$ is relatively conservative, which is mainly determined by using the average braking deceleration 0.4g based on NHTSA statistical results, the current vehicle speed and the driver response time, the warning state threshold time at different vehicle speeds are shown in Fig 7(a).

The vehicle emergency braking threshold time $T_{th3}$ and $T_{th4}$ are determined by the vehicle braking distance Equation (22) and the improved TTC model Equation (15). Assuming the current vehicle speed is $v_{\text{0}}$, the braking distance of the vehicle can be calculated through Equation (22), and the collision time can be obtained according to Equation (15). Finally, by setting different initial vehicle speed $v_{\text{0}}$ and relative vehicle speed $v_{r}$, and using linear interpolation method, the three-dimensional map of $T_{th3}$ under different working conditions is obtained, which as shown in the Fig 7(b). Similarly, set the braking deceleration of full braking to −0.6g, the threshold time map $T_{th4}$ can be obtained, which as shown in Fig 7(c).

(3) State threshold time correction based road adhesion characteristics

The above state threshold time calculation process ignores the influence of road adhesion coefficient. In the process of emergency braking, the vehicle's maximum braking deceleration depends on the tire-pavement maximum adhesive force. In order to improve the working condition adaptability of the AEB control strategy under different road surface adhesion coefficients, the state threshold time is corrected as:


$$\begin{array}{c} {T^{\prime }}_{thi}=\left\{ \begin{array}{c} @l@T_{thi}\text{\textbackslash{}hspace\{1em}\;\;\;\}\mu \geq 0.6\\ T_{thi}\frac{0.6}{\mu }\text{\textbackslash{}hspace\{1em}\;\;\;\}\mu <0.6 \end{array}\right.\text{\textbackslash{}hspace\{1em}\}i=1,2,3,4 \end{array}$$ (23)

where, μ is the road adhesion coefficient. When μ≥0.6, the road surface can provide the adhesive force required for maximum braking deceleration to −0.6g, and the time threshold is not adjusted at this time. Otherwise, when μ<0.6, the road surface cannot provide the adhesive force required for full braking of the vehicle, and the state threshold time should be corrected in real time based on changes in road surface adhesion coefficient. As the road adhesion coefficient decreases, the timing of AEB warning and braking intervention is advanced, thereby fully ensuring the safety of vehicles on low adhesion roads such as wet, slippery, icy, and snowy.

## 4. Design of AEB hierarchical control strategy

**Fig 8:**
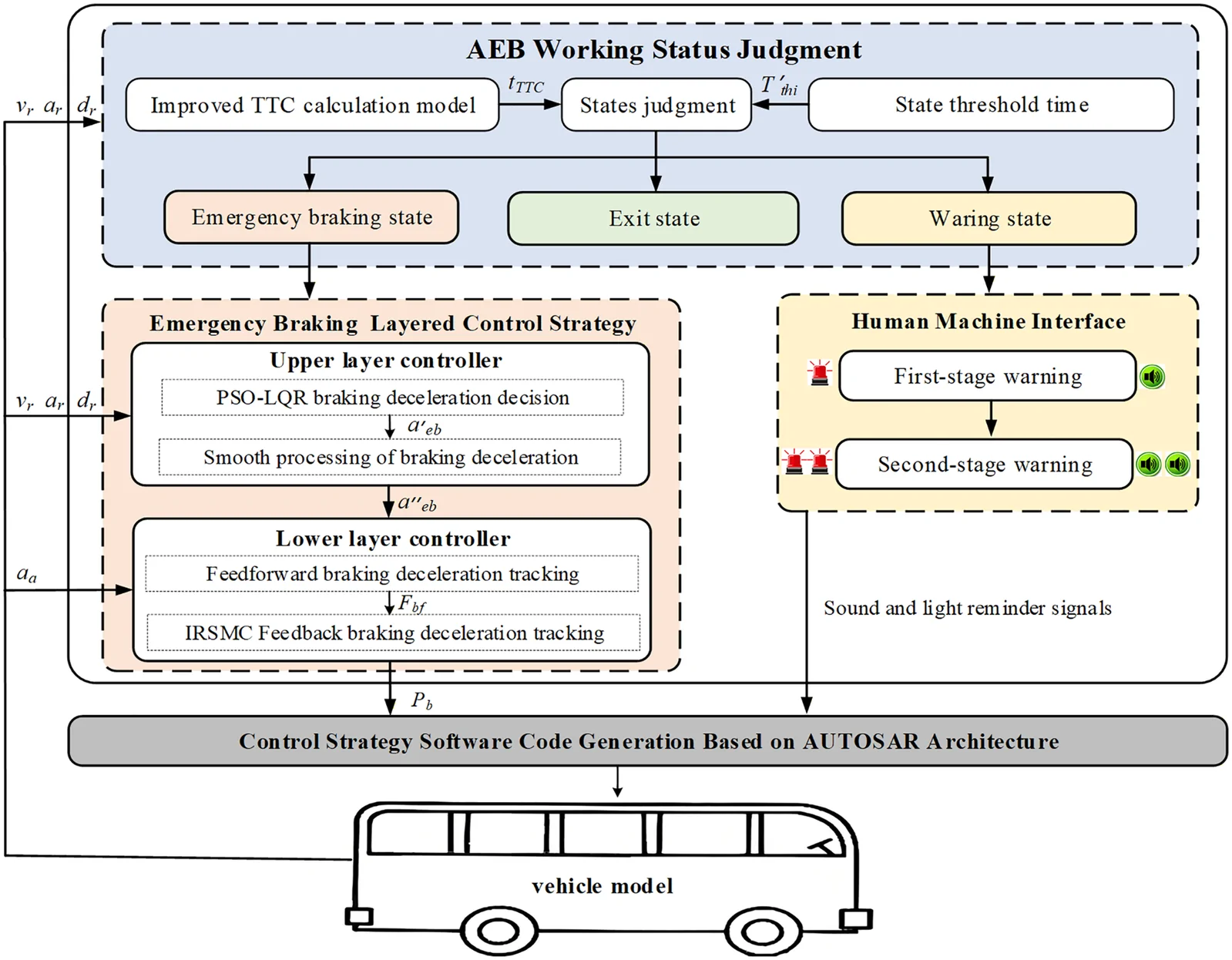
Overall framework of AEB hierarchical control strategy.

According to the emergency collision avoidance function requirements of the AEB system, a hierarchical control concept is adopted to develop the overall framework of emergency braking control strategy, as shown in Fig 8. The upper layer controller is an expected braking deceleration optimal decision controller. Based on the inter-vehicle's motion state model, a PSO-LQR controller is designed to obtain expected braking deceleration, which takes into account both vehicle safety and comfort. The lower layer controller is the expected braking deceleration robust tracking controller, which uses feedforward control to obtain the required braking force to enhance the tracking speed of the expected braking deceleration, and improves the tracking accuracy through real-time feedback control. Finally, an AEB software architecture based on AUTOSAR is designed. The in-vehicle environment sensing sensor measurement accuracy, vehicle mass and road state parameters have a significant impact on the control effect and robustness of the proposed AEB strategy. It should to be noted that due to the sensor signal processing and driving condition parameter estimation is not the focus of this paper, the state parameter information involved in AEB control strategy is assumed to be known in real time, which has not been studied in-depth

### 4.1. The upper layer expected braking deceleration optimal decision controller

(1) The motion relationship model between inter-vehicle

**Fig 9:**
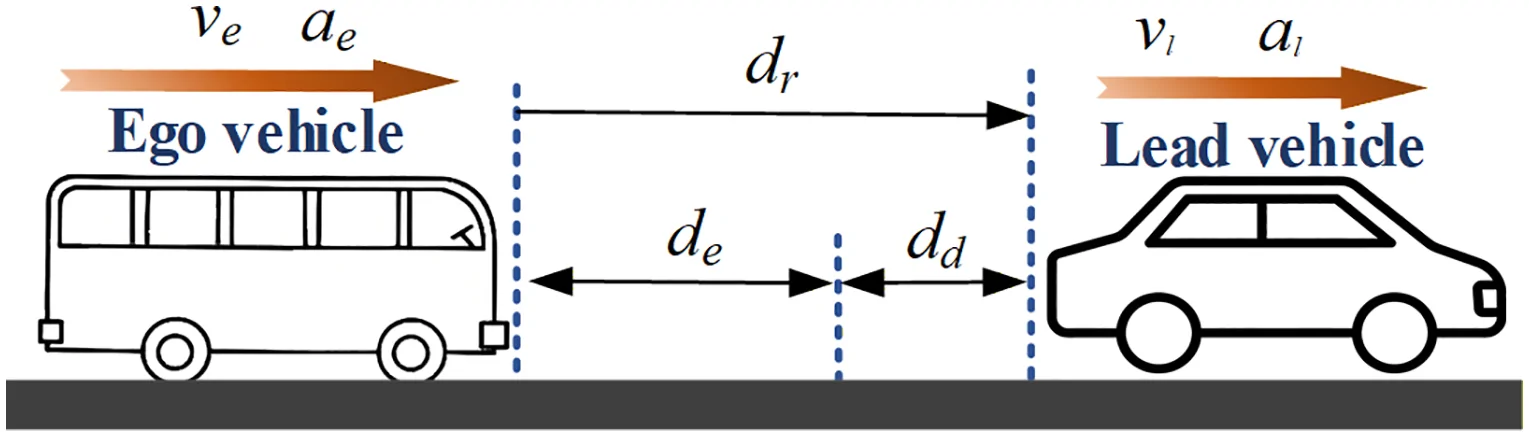
The motion relationship between inter-vehicle.

The AEB system needs to make actions according to the relative motion state between the ego vehicle and the lead vehicle, so the longitudinal motion model between inter-vehicle is the basis for determining the expected braking deceleration. During the driving process, the longitudinal motion relationship between inter-vehicle is displayed in Fig 9.

From in Fig 9, the longitudinal motion relationship between the front and rear vehicles can be expressed as:


$$\begin{array}{c} \left\{ \begin{array}{c} @l@d_{d}=d_{r}-d_{e}\\ d_{e}=\tau_{h}v_{e}+d_{\text{0}}\\ v_{r}=v_{l}-v_{e}\\ a_{r}=a_{l}-a_{e} \end{array}\right. \end{array}$$ (24)

where, $d_{e}$ is the expected distance between inter-vehicle; $d_{d}$ denotes the deviation distance between inter-vehicle; $d_{\text{0}}$ represents the minimum safety distance, $d_{\text{0}}=3m$; $t_{h}$ represents the driver style time constant.

(2) Design of PSO-LQR expected braking deceleration controller

In the upper layer controller of AEB control strategy, a linear quadratic regulator (LQR) is used to achieve the expected braking deceleration adaptive decision. The LQR control algorithm can achieve optimal control of system performance by minimizing the preset performance indicators, which has the ease of implementation and strong robustness [33]. Firstly, based on the motion relationship between inter-vehicle as displayed in Equation (24), the state equation of the AEB control system is built, which can be expressed as:


$$\begin{array}{c} \left\{ \begin{array}{c} @l@\dot{X}\left(t\right)=𝐴X\left(t\right)+𝐵U\left(t\right)\\ Y\left(t\right)=𝐶X\left(t\right) \end{array}\right. \end{array}$$ (25)

where, $X\left(t\right)=[d_{d},\text{\textbackslash{}hspace\{0.33em}\}v_{r},\text{\textbackslash{}hspace\{0.33em}\}a_{r}{]}^{T}$, which represents the system state variables; Y(t) is the system output variables; U(t) denotes the system control variables, which is the vehicle’s expected braking deceleration $a_{eb}$. Due to the response time of the braking system, the vehicle’s actual braking deceleration usually lags behind the expected braking deceleration, which can be approximately simplified into a first-order inertia function.


$$\begin{array}{c} {a^{\prime }}_{eb}=\frac{\text{1}}{T_{l}s+1}a_{eb} \end{array}$$ (26)

where, $T_{l}$ is the time constant.

Therefore, the coefficients 𝐴, 𝐵, and 𝐶 of the state Equation (25) can be expressed as follows:


$$\begin{array}{c} A=[\begin{array}{ccc} 0 & 1 & -\tau_{\text{h}}\\ 0 & 0 & -1\\ 0 & 0 & -1/T_{l} \end{array}],B=[\begin{array}{c} 0\\ 0\\ 1/T_{l} \end{array}],C=[\begin{array}{ccc} 1 & 0 & 0\\ 0 & 1 & 0\\ 0 & 0 & 1 \end{array}] \end{array}$$ (27)

According to the system state equation controllability judgment matrix $M=[𝐵\text{\textbackslash{}hspace\{0.33em}\}𝐴𝐵\cdot \cdot \cdot {𝐴}^{\text{n-1}}𝐵{\text{]}}^{\text{T}}\text{(n=3)}$, can be concluded rank(M)=3, that is the system is controllable. At the same time, according to the system state equation observability judgment matrix $N=[𝐶\text{\textbackslash{}hspace\{0.33em}\}𝐶𝐴\cdot \cdot \cdot 𝐶{𝐴}^{\text{n-1}}{\text{]}}^{\text{T}}\text{(n=3)}$, can be seen rank(N)=3, that is the system is observable. Therefore, there exists an optimal value so that the following cost function is minimized.


$$\begin{array}{c} J=\frac{\text{1}}{\text{2}}\int_{\text{0}}^{\infty }\left[x(t{)}^{\text{T}}Qx(t)+u(t{)}^{\text{T}}Ru\left(t\right)\right]dt \end{array}$$ (28)

where, Q and R are the weight coefficient matrices of state variables and control variables, respectively, which can be expressed as:


$$\begin{array}{c} 𝐐=\left[\begin{array}{ccc} q_{\text{1}} & \text{0} & 0\\ 0 & q_{\text{2}} & \text{0}\\ 0 & 0 & q_{\text{3}} \end{array}\right],R=r \end{array}$$ (29)

In the AEB control strategy, the cost function of LQR comprehensively considers safety and comfort. The safety cost function comprehensively considers the vehicle's relative distance $d_{d}$, relative speed $v_{r}$, and relative braking deceleration $a_{r}$, which can be expressed as:


$$\begin{array}{c} J_{\text{1}}=\int_{\text{0}}^{\infty }X^{T}QXdt=\int_{\text{0}}^{\infty }\left[q_{\text{1}}(d_{d}-d_{ref}{)}^{\text{2}}+q_{\text{2}}(v_{r}-v_{ref}{)}^{\text{2}}+q_{\text{3}}(a_{r}-a_{ref}{)}^{\text{2}}\right]dt \end{array}$$ (30)

where, $q_{\text{1}}$, $q_{\text{2}}$, $q_{\text{3}}$, and r are the weight coefficients;$d_{ref}$, $v_{ref}$, $a_{ref}$ are the reference value of the control target, to ensure collision avoidance safety, $d_{ref}=0$, $v_{ref}=0$, $a_{ref}=0$.

Therefore, the Equation (1.4) can be simplified as:


$$\begin{array}{c} J_{\text{1}}=\int_{\text{0}}^{\infty }X^{T}QXdt=\int_{\text{0}}^{\infty }\left[q_{\text{1}}{d_{d}}^{\text{2}}+q_{\text{2}}{v_{r}}^{\text{2}}+q_{\text{3}}{a_{r}}^{\text{2}}\right]dt \end{array}$$ (31)

The goal of LQR control is to make $J_{\text{1}}$ approach zero $J_{\text{1}}\to 0$ or reach its minimum value. When the vehicle achieves emergency braking function, the relative distance, relative velocity, and relative braking deceleration both tend to zero, that is, $d_{d}\to 0$, $v_{r}\to 0$, $v_{r}\to 0$. Therefore, when $J_{\text{1}}$ approaches its minimum value, the vehicle can quickly achieve emergency collision avoidance function and improve the active safety.

The main consideration for braking comfort is to minimize the change of LQR control law u(t), that is, the expected braking deceleration is minimum. The braking ride comfort cost function can be expressed as:


$$\begin{array}{c} J_{\text{2}}=\frac{\text{1}}{\text{2}}\int_{\text{0}}^{\infty }r{a^{\prime }}_{eb}^{\text{2}}dt \end{array}$$ (32)

where, r is the weight coefficients.

The expected braking deceleration is minimal, which can reduce the maximum longitudinal jerk of the vehicle during emergency braking, thereby improving the ride comfort.

Comprehensive consideration of vehicle safety and braking ride comfort, the cost function of LQR can be expressed as:


$$\begin{array}{c} J=J_{\text{1}}+J_{\text{2}}=\frac{\text{1}}{\text{2}}\int_{\text{0}}^{\infty }\left[q_{\text{1}}(d_{d}-d_{ref}{)}^{\text{2}}+q_{\text{2}}(v_{r}-v_{ref}{)}^{\text{2}}+q_{\text{3}}(a_{r}-a_{ref}{)}^{\text{2}}+r{a^{\prime }}_{eb}^{\text{2}}\right]dt \end{array}$$ (33)

Design the feedback gain coefficient matrix K to minimize the cost function J of the control system, set U(t)=−KX(t), the feedback gain coefficient matrix can be calculated as:


$$\begin{array}{c} K=R^{-1}{𝐵}^{T}Z \end{array}$$ (34)

where, Z is the solution of Riccati differential equations. The optimal expected braking deceleration can be expressed as:


$$\begin{array}{c} {a^{\prime }}_{eb}=U\left(t\right)=-KX\left(t\right) \end{array}$$ (35)

From the above-mentioned design process of the LQR controller, it can be seen that the optimal feedback gain coefficient matrix K of the controller depends on the weight matrix Q and R. However, the current method of determining the value of Q and R generally depends on the designer's experience, which would consume a lot of time and effort on parameter adjustment, and even further lead to the LQR controller not achieving the best control effect.

**Fig 10:**
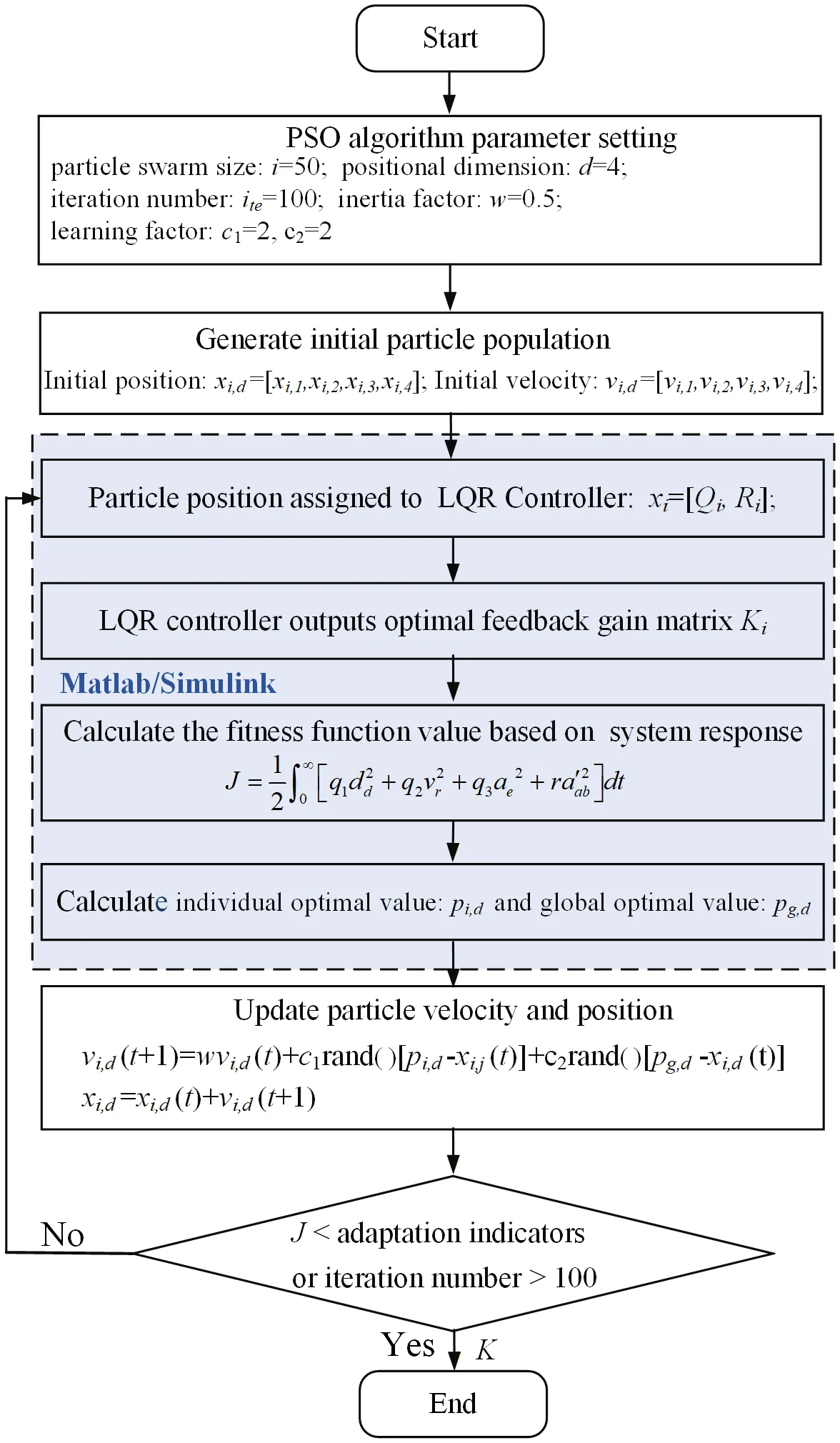
Schematic of PSO algorithm optimized design of LQR controller.

To solve this problem, this paper utilizes the global traversal optimization capability of particle swarm optimization algorithm [34], a method for PSO optimization of LQR controller is proposed. The weight coefficient matrices Q and R for achieving the best control effect of the vehicle during emergency braking control are obtained through PSO optimization, thereby eliminating the subjective influence of experience on traditional LQR controller. The design process of PSO-LQR controller is shown in Fig 10.

(3) Smooth processing of expected braking deceleration considering comfort

At the moment when the AEB system triggers emergency braking from a warning state, there may be a sudden increase in the expected braking deceleration determined by PSO-LQR, resulting in a decrease in the braking comfort of the vehicle. Therefore, two methods are adopted to optimize the expected braking deceleration value.

The first method is to limit the amplitude. To prevent excessive expected braking deceleration, referring the maximum expected braking deceleration is set to $a_{2b}=-0.6g$ based on the traditional DW-SB control strategy. The expected braking deceleration after amplitude limiting processing can be re-expressed as:


$$\begin{array}{c} {a^{\prime \prime }}_{eb}=\left\{ \begin{array}{c} @l@\;{a^{\prime }}_{eb},\;\;\left|a_{eb}\right|\leq 0.6g\&\left|a_{eb}\right|\leq \mu g\text{\textbackslash{}hspace\{0.33em}\}\mathrm{\backslash vspace}1mm\\ -0.6g,\;\;\;\left|a_{eb}\right|>0.6g\&\left|a_{eb}\right|\leq \mu g\;\\ -\mu \text{g\textbackslash{}hspace\{0.33em}\;\;\;else\} \end{array}\right. \end{array}$$ (36)

Moreover, refer to the research results of Kyongsu [35], when the vehicle's deceleration rate is less than 10m/s^3^, the comfort during braking can be effectively guaranteed. Therefore, the change rate of the expected braking deceleration is subject to the following constraints:


$$\begin{array}{c} \left|\frac{\text{d}{a^{\prime \prime }}_{eb}}{dt}\right|\leq 10\text{\textbackslash{}hspace\{0.17em}\}m/s^{\text{3}} \end{array}$$ (37)

**Fig 11:**
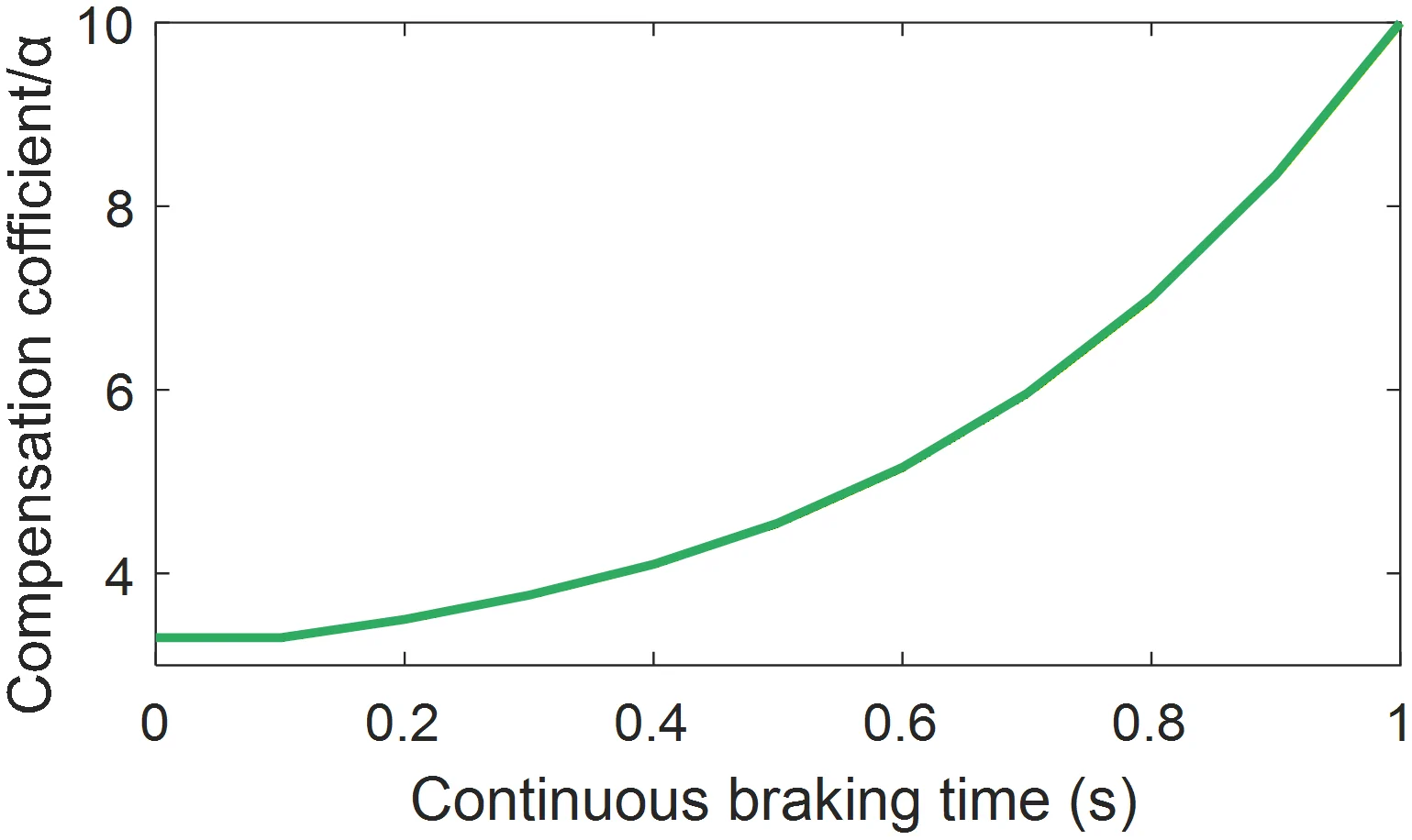
Time compensation coefficient curve.

The second method is to smooth correction. To prevent the excessive change rate of expected braking deceleration from affecting the comfort during emergency braking, a time compensation coefficient is introduced to smooth the expected braking deceleration curve based on PSO-LQR controller.


$$\begin{array}{c} {a^{\prime \prime }}_{eb}=(\text{\textbackslash{}hspace\{0.17em}\}1-e^{-\alpha t}\text{\textbackslash{}hspace\{0.17em}\})\text{\textbackslash{}hspace\{0.17em}\}{a^{\prime }}_{eb} \end{array}$$ (38)

where, t is the continuous braking time; α denotes the time compensation coefficient, it can effectively improve sudden changes in braking deceleration, thereby enhancing ride comfort. α is increases with the increase of t, and its changing trend is shown in Fig 11.

### 4.2. The lower layer expected braking deceleration robust tracking controller

The lower layer controller of the AEB control strategy system mainly controls the braking system, so that the vehicle’s actual braking deceleration can be well tracked the expected braking deceleration decided by the upper layer controller.

(1) Feedforward controller design

To improve the response speed of vehicle braking deceleration, a feedforward open-loop controller is designed. According to the longitudinal dynamic equation of vehicle driving, the required braking force when the expected braking deceleration is ${a^{\prime \prime }}_{eb}$ can be expressed as:


$$\begin{array}{c} F_{bf}=m{a^{\prime \prime }}_{eb}+mgsin\beta +C_{D}A\rho v^{\text{2}}/2+mgf \end{array}$$ (39)

(2) Feedback controller design

During the braking process, the vehicle will be disturbed by various factors such as road adhesion coefficient changes and load transfer. To improve the accuracy and anti-interference performance of expected braking deceleration tracking, a robust integral sliding mode control (RISMC) strategy is designed.

In order to quickly and smoothly reach the balance point after the control system enters the sliding mode, the expected braking deceleration tracking error is selected as the sliding mode surface function. On this basis, an integral term for control error is added to eliminate the steady-state error of the system, and the sliding mode surface function can be expressed as:


$$\begin{array}{c} s={a^{\prime \prime }}_{eb}-a_{b}+c\int ({a^{\prime \prime }}_{eb}-a_{b}) \end{array}$$ (40)

where, $a_{b}$ denotes the vehicle’s actual braking deceleration, c is the integral term constant.

By taking the derivative of the sliding surface function, it can be obtained:


$$\begin{array}{c} \dot{s}=-{\dot{a}}_{b}+c{a^{\prime \prime }}_{eb}-ca_{b} \end{array}$$ (41)

According to the longitudinal dynamic balance equation of the vehicle, the braking deceleration can be calculated as:


$$\begin{array}{c} \left\{ \begin{array}{c} @l@a_{b}=\dot{v}=-gsin\beta -gf-\frac{C_{D}A\rho v^{\text{2}}}{\text{2}}+\frac{F_{b}}{m}=C_{v}+\frac{F_{b}}{m}\\ C_{v}=-gsin\beta -gf-\frac{C_{D}A\rho v^{\text{2}}}{\text{2}} \end{array}\right. \end{array}$$ (42)

By combining Equation (41) and Equation (42), and the system disturbance and uncertainty are comprehensively considered, it can be obtained as:


$$\begin{array}{c} \dot{s}=C_{v}\left(C_{D}A\rho v/m-c\right)-F_{b}\left(c+C_{D}A\rho v/m\right)/m+c{a^{\prime \prime }}_{eb}+D \end{array}$$ (43)

where, D is a bounded disturbance, that is, $\left|D\right|\leq D_{b}$.

In order to quickly approach and maintain the system state on the sliding surface of the controller, and improve the response speed of the controller, an exponential approach law is adopted as the sliding surface approach function, which can be expressed as:


$$\begin{array}{c} \left\{ \begin{array}{c} @l@\dot{s}=-\varepsilon sgn\left(s\right)-ks\text{\textbackslash{}hspace\{0.33em}\;\}\varepsilon >0,k>0\\ sgn\left(s\right)=\left\{ \begin{array}{c} @l@1\text{\textbackslash{}hspace\{1em}\;\;\;\}s>0\\ 0\text{\textbackslash{}hspace\{1em}\;\;\}\;s=0\\ -1\text{\textbackslash{}hspace\{1em}\;\}\;s<1 \end{array}\right. \end{array}\right. \end{array}$$ (44)

Combining Equation (43) and Equation (44), it can be obtained:


$$\begin{array}{c} F_{bb}=\frac{m^{\text{2}}\left(c{a^{\prime \prime }}_{eb}+\varepsilon sgn\left(s\right)+ks\right)}{mc+C_{D}A\rho v}+mC_{v} \end{array}$$ (45)

To verify the stability of the RISMC control system, a Lyapunov function is constructed as follows:


$$\begin{array}{c} \lambda =\frac{\text{1}}{\text{2}}s^{\text{2}} \end{array}$$ (46)

By taking the first derivative of the Lyapunov function mentioned above, it can be obtained:


$$\begin{array}{c} \dot{\lambda }=s\dot{s}=s[D-\varepsilon sgn(s)-ks]\leq -\left|s\right|(\text{\textbackslash{}hspace\{0.17em}\}\varepsilon -D) \end{array}$$ (47)

Therefore, when $\varepsilon >D_{b}$, $\dot{\lambda }<0$, the control system is stable. However, the coefficient ε of the discontinuous sign function is too large, it will easily cause chattering problems in the control system. To solve this problem, the saturation function is used to replace the traditional sign function to suppress the chattering of the system, which can be expressed as:


$$\begin{array}{c} sat\left(s\right)=\left\{ \begin{array}{c} @l@s\text{\textbackslash{}hspace\{1em}\;\;\;\;\}\left|s\right|<1\\ sgn\left(s\right)\left|s\right|\geq 1 \end{array}\right. \end{array}$$ (48)

The Equation (48) can be re-expressed as:


$$\begin{array}{c} F_{bb}=\frac{m^{\text{2}}\left(c{a^{\prime \prime }}_{eb}+\varepsilon sat\left(s\right)+ks\right)}{mc+C_{D}A\rho v}+mC_{v} \end{array}$$ (49)

By combining feedforward control and feedback control, the total required braking force of the vehicle can be ultimately expressed as:


$$\begin{array}{c} F_{eb}=F_{bf}+F_{bb} \end{array}$$ (50)

**Fig 12:**
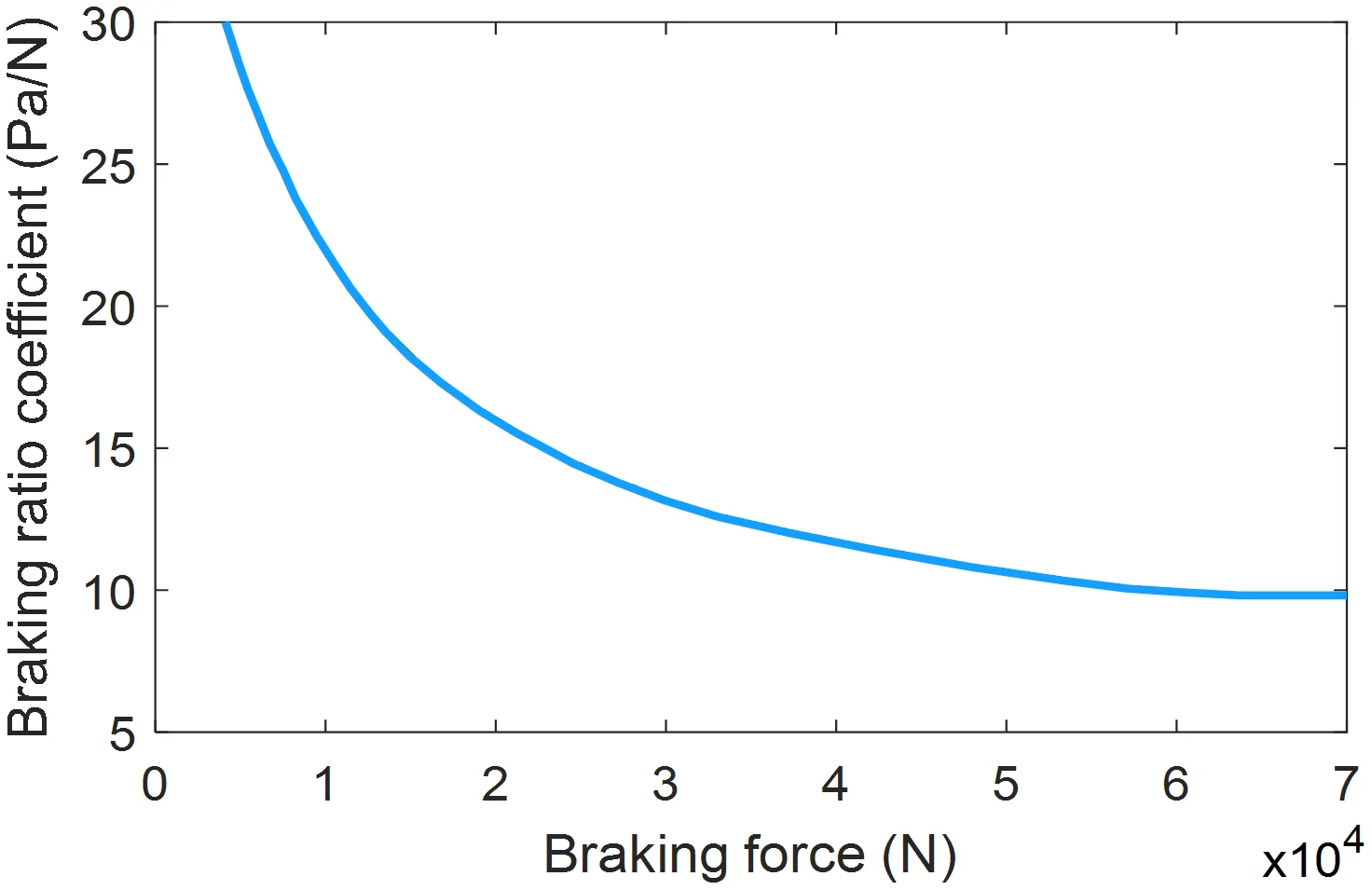
Braking ratio coefficient curve.

When performing braking control, the required braking force signal of the vehicle needs to be converted into the required braking pressure signal of the braking system, and the conversion relationship can be expressed as:


$$\begin{array}{c} F_{eb}=K_{b}P_{b} \end{array}$$ (51)

where, $P_{b}$ denotes the braking system pressure; $K_{b}$ is braking ratio coefficient, it can be represented as shown in the Fig 12.

### 4.3. AEB control strategy software architecture

**Fig 13:**
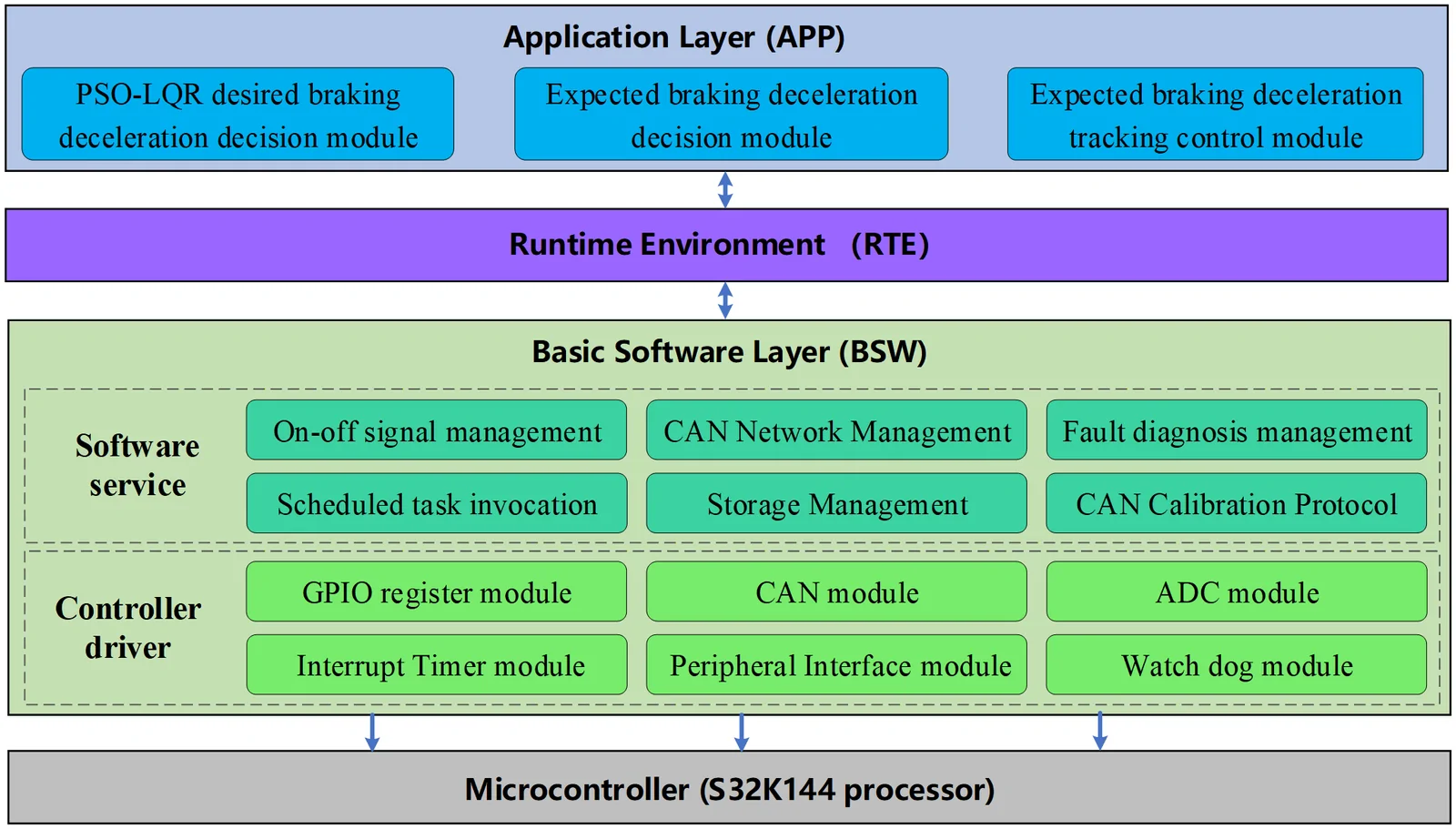
AEB control strategy software architecture based on AUTOSAR.

In order to enhance the quality of AEB control strategy software code generation, improve the portability and standardization of ECU software, an AEB software based on Automotive Open System Architecture (AUTOSAR) has been designed, which as shown in Fig 13.

The AEB software architecture is divided into three parts: application layer (APP), runtime environment layer (RTE), and basic software layer (BSW). The APP is the core of the AEB software and the key to achieving emergency collision avoidance functionality, which primarily consists of the AEB control strategy established using MATLAB/Simulink software. The RTE is responsible for managing and coordinating the information interaction between APP and BSW, it realizes the decoupling between the lower BSW and the upper APP by providing a unified communication interface. The BSW provides underlying basic software driver functions and services. It provides support and interfaces for the interaction between upper layer APP and microcontroller hardware. The microcontroller of the AEB control system is S32K144, and the software development environment is S32 Design Studio. The AEB software in the controller is downloaded and upgraded through the design of the BootLoader program and the CAN bus interface.

## 5. Hardware-in-the-loop and real vehicle experiments

### 5.1. Hardware-in-the-Loop simulation test

To verify the control effect of the proposed AEB control strategy, a Hardware-in-the-Loop (HiL) simulation test platform based on NI/PXI is built. The HiL test platform is mainly composed of controller hardware and upper computer software. The controller hardware is mainly composed of NI/PXI real-time simulator, AEB control unit, CANoe and voltage-regulating power supply. The upper computer software is mainly composed of NI Veristand, TruckSim and MATLAB/Simulink. The NI/PXI real-time simulator is used to connect the AEB control unit with the Trucksim software simulation test scenarios, and the NI Veristand is responsible for calibrating control strategy parameters and collecting data. The vehicle parameters are shown in Table 1.

**Table 1:** Commercial buses parameters.

Parameters	Value	Parameters	Value
Vehicle mass (*m*/kg)	7960	Distance from hitch to front axle (*l*_f_/mm)	0.33
Center of mass height (*h*/mm)	1200	Front area (*A*_w_/m^2^)	510
Wheel track (*b*/mm)	2050	Tire radius (*R*_w_/mm)	8.4
Wheel base (*l*/mm)	4490	Air drag coefficient (*C*_w_)	3105

Referring to the requirements of the European New Car Assessment Programme (E-NCAP) for AEB functional testing procedures [36], this paper selects three typical application scenarios: Car-to-Car Rear Stationary (CCRs), Car-to-Car Rear Moving (CCRm) and Car-to-Car Rear Braking (CCRb) to verify the effectiveness of the developed AEB control strategy.

(1) CCRs

**Fig 14:**
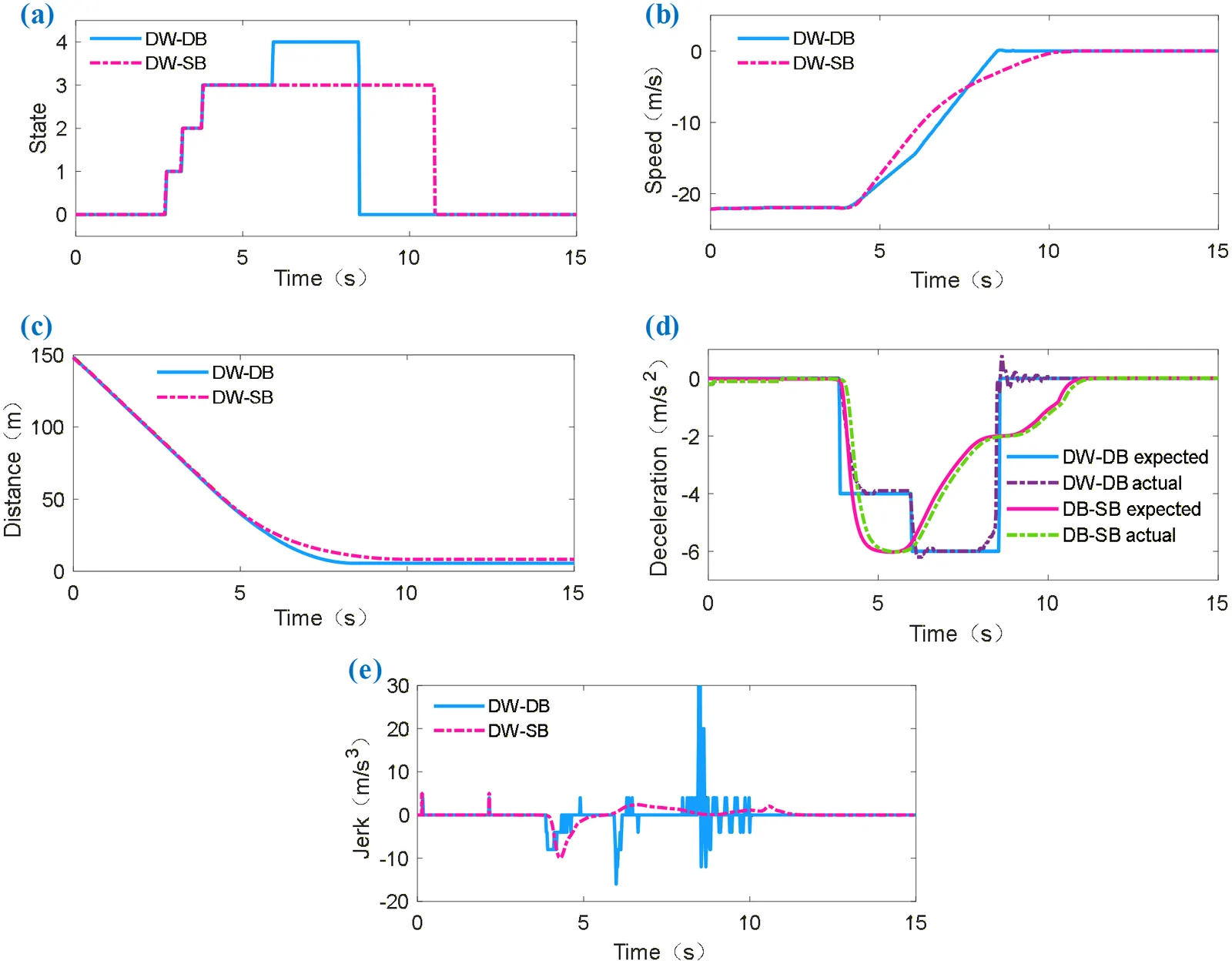
CCRs working condition simulation results. (a) working state. (b) inter-vehicle relative speed. (c) inter-vehicle relative distance. (d) braking deceleration. (e) longitudinal jerk.

The simulation conditions are set as follows: the ego vehicle driving in a straight line at a speed of 80 km/h (22.2m/s), the lead vehicle remains stationary, and the initial inter-vehicle relative distance is set to 150m. The simulation results of CCRs working condition are shown in Fig 14.

As shown in Fig 14(a), two AEB control strategies enter together the first-stage warning and second-stage warning state at 2.7s and 3.18s. As the inter-vehicle relative distance decreases, the collision risk increases, and finally enter the emergency braking state at 3.78s. In Figs 14(b) and (c), the traditional DW-DB control strategy and the proposed DW-SB control strategy reduce the inter-vehicle relative speed to 0m/s at 8.46s and 10.73s respectively, that is, the ego vehicle stops by emergency braking. At this time, the inter-vehicle relative distance is 5.72m and 8.33m respectively, and the AEB system exits the emergency braking state. As displayed in Fig 14(d), under the two control strategies, the vehicle’s actual braking deceleration can track the expected braking deceleration well, but the proposed DW-SB control strategy makes the expected braking deceleration change smoother. From Fig 14(e), the maximum longitudinal jerk (braking deceleration change rate) of the vehicle under the two strategies is 30 m/s^3^ and 9.95 m/s^3^ respectively. The proposed DW-SB control strategy has decreased by 66.83% compared to traditional DW-DB, indicating that the longitudinal jerk of the vehicle is smaller during the emergency braking process of AEB.

Through the analysis of the simulation results in Fig 14, it can be concluded that both the DW-DB control strategy and DW-SB control strategy can achieve the vehicle’s emergency collision avoidance function under CCRs working condition. Although the DW-DB takes a shorter time to achieve emergency braking, its expected braking deceleration cannot be adaptively adjusted according to the actual conditions, resulting in a large vehicle’s longitudinal jerk and poor braking comfort. The proposed DW-SB can adaptively adjust the excepted braking deceleration in real time according to the vehicle's driving state information, so that the deceleration change rate is smoother, which has better driving and ride comfort during emergency braking.

(2) CCRm

**Fig 15:**
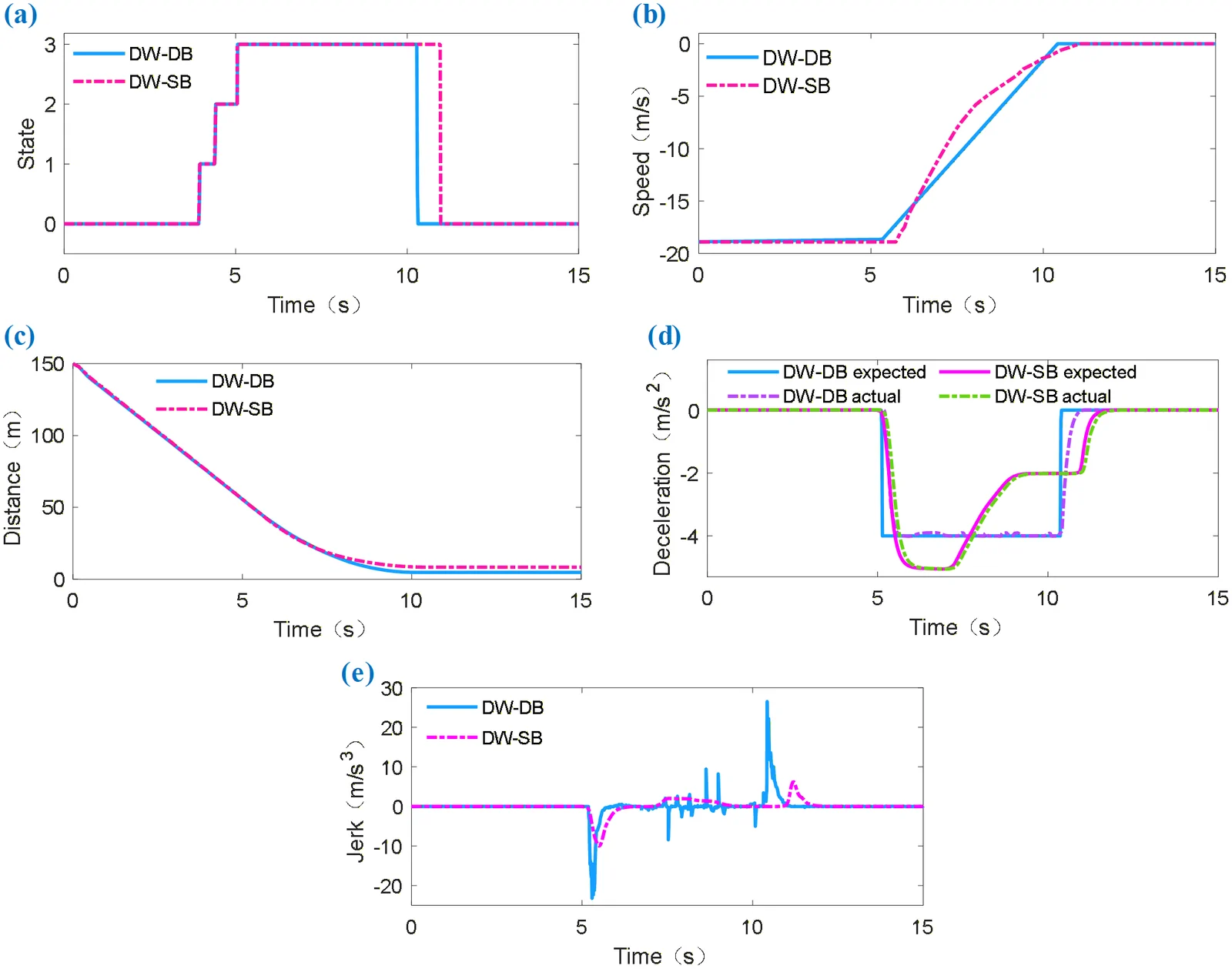
CCRm working condition simulation results. (a) working state. (b) inter-vehicle relative speed. (c) inter-vehicle relative distance. (d) braking deceleration. (e) longitudinal jerk.

The simulation conditions are set as follows: the ego vehicle driving in a straight line at a speed of 80 km/h (25m/s), the lead vehicle driving at a constant speed of 12 km/h (3.3m/s), and the initial inter-vehicle relative distance is set to 150m. The simulation results of CCRm working condition are shown in Fig 15.

In Fig 15(a), the traditional DW-DB control strategy enters the emergency braking state at 5.08 seconds, but does not trigger the full braking state, the DW-DB and DW-SB exit the emergency braking state at 10.32s and 10.98s respectively. As depicted in Figs 15(b) and (c), when the DW-DB and DW-SB control strategies exit the emergency braking state, the inter-vehicle relative speed is maintained at 0m/s, and the inter-vehicle relative distance is 4.7m and 8.3m respectively. It indicates that the emergency braking distance of DW-SB is shorter, and the motion state of the ego and lead vehicle is safer. As displayed in Fig 15(d), under the two control strategies, the vehicle’s actual deceleration can track the expected braking deceleration well, but the expected braking deceleration curve determined by the DW-SB is smoother. From Fig 15(e), the maximum longitudinal jerk of the vehicle under the two strategies are 25.1m/s^3^ and 9.91m/s^3^ respectively, and the proposed DW-SB control strategy has decreased by 60.52% compared to traditional DW-DB.

In summary, under CCRm working condition, both DW-DB and DW-SB can achieve the vehicle’s emergency collision avoidance function, and the DW-SB control strategy proposed in this paper can keep a longer inter-vehicle safe distance while providing better braking comfort.

(3) CCRb

**Fig 16:**
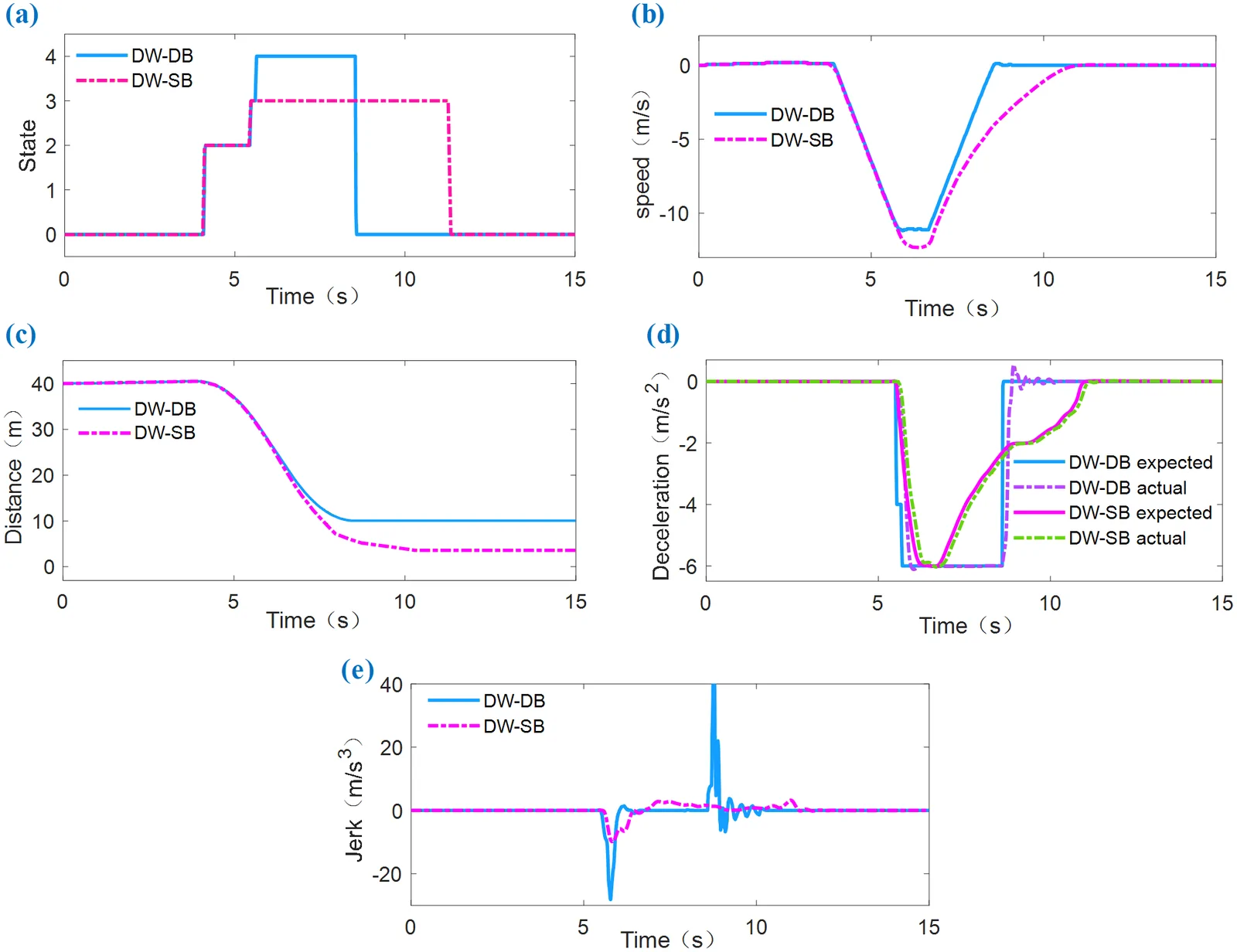
CCRb working condition simulation results. (a) working state. (b) inter-vehicle relative speed. (c) inter-vehicle relative distance. (c) inter-vehicle relative distance. (e) longitudinal jerk.

The simulation conditions are set as follows: the ego vehicle driving in a straight line at a speed of 80 km/h (25m/s), the initial inter-vehicle relative distance is set to 40m; the lead vehicle is driving at an initial speed of 80 km/h, and at the 4th second, the deceleration changes to -6m/s^2^. The simulation results of CCRb working condition are shown in Fig 16.

As shown in Fig 16(a), due to the short inter-vehicle relative distance, the collision risk level of the vehicle is relatively high when the lead vehicle enters the braking deceleration state, the AEB system directly enters the second level warning state at 4.05s and triggers the emergency braking state at 5.42 seconds. The traditional DW-DB AEB control strategy enters full braking state at 5.62 seconds, and exits at 8.58 seconds. The DW-DB and DW-SB exited the emergency braking state at 8.58s and 11.35 s, the duration of the entire braking process is 3.16s and 5.93s respectively. In Figs 16(b) and (c), when the AEB control system exited, the vehicle speed has decreased to 0m/s, the inter-vehicle relative distances are 10.8m and 3.6m respectively, and the vehicle does not collide. As described in Figs 16(d) and (e), when performing emergency braking control, the vehicle’s actual braking deceleration can track the expected braking deceleration well under two AEB control strategies. The maximum longitudinal jerks of the vehicle are 40 m/s^3^ and 9.81 m/s^3^ respectively, and the proposed DW-SB control strategy has decreased by 75.5% compared to traditional DW-DB.

In summary, under CCRb working condition, both DW-DB and DW-SB can achieve the vehicle’s emergency collision avoidance function. The proposed DW-DB control strategy has a longer emergency braking working time, but the braking deceleration changes more smoothy, the vehicle’s longitudinal jerk is smaller and the braking comfort is better compared with traditional DW-DB.

### 5.2. DW-DB control strategy real vehicle experiment

To verify the engineering practicality of the proposed DW-SB control strategy in this paper, a 12-meter-long and 39 seats commercial buses is used as the experimental vehicle. Based on the original drive/braking system of the vehicle, the emergency braking function is achieved by adding camera, millimeter wave radar and AEB controller.

**Fig 17:**
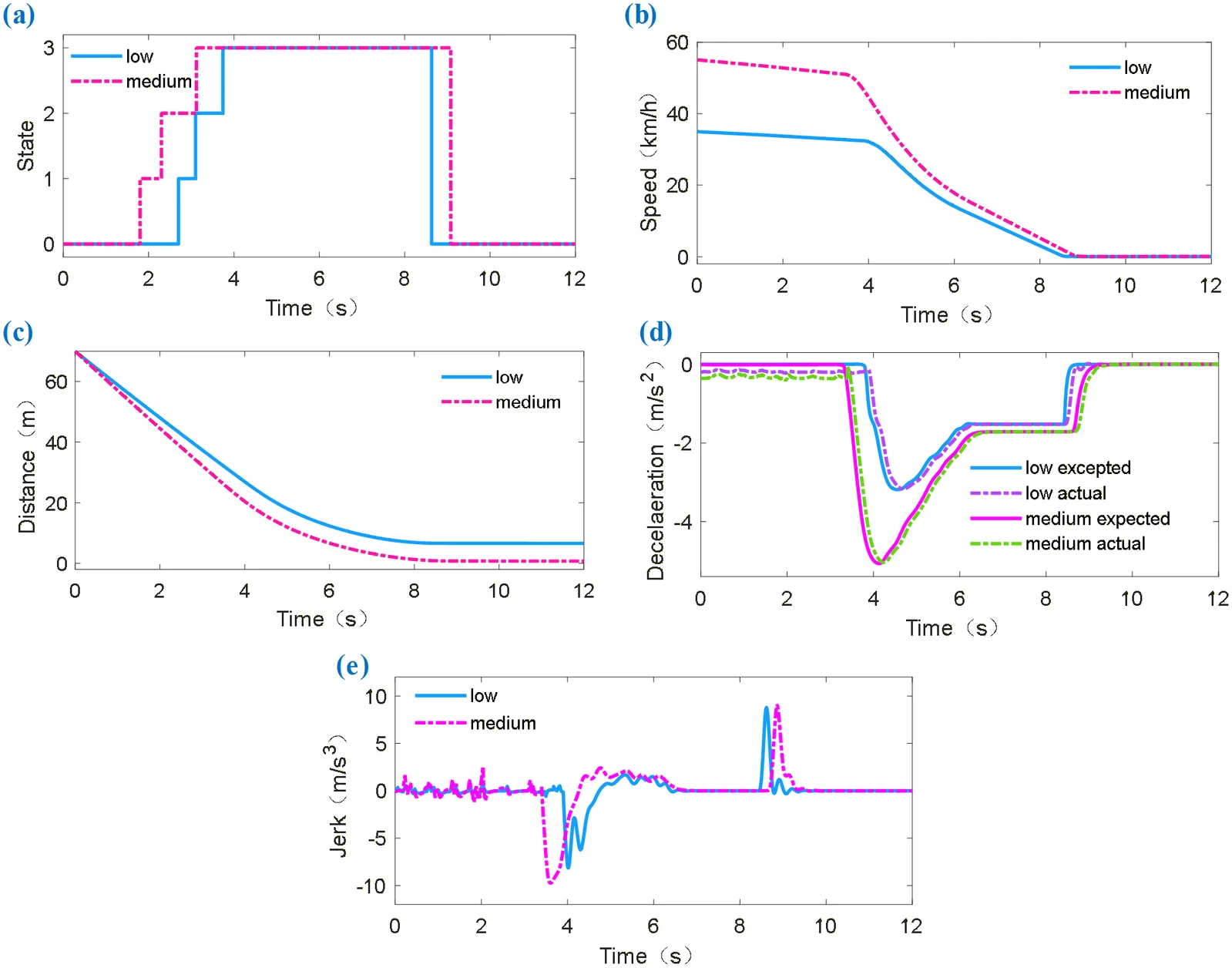
Real vehicle experiment results. (a) working state. (b) ego vehicle speed. (c) inter-vehicle relative distance. (d) braking deceleration. (e) longitudinal jerk.

Referring to the maximum vehicle speed limit of urban roads, which usually does not exceed 60 km/h. The actual vehicle experiment conditions in this paper are set as: the lead vehicle remains stationary, the ego vehicle is driving at a low-speed of 35 km/h and a medium-speed of 55 km/h respectively, and the initial inter-vehicle distance is set to 70m. The real vehicle experiment results are shown in Fig 17.

As displayed in Fig 17(a), under low-speed and medium-speed experiment conditions, the AEB control system enters emergency braking state at 3.74s and 3.1s respectively, and the braking duration at high-speed is longer than at low-speed. In Fig 17(b), due to the presence of driving resistance, the vehicle appeared some speed loss when emergency braking is not applied. In Figs 17(b) and (c), when the vehicle exits the emergency braking state, its speed decreases to 0 km/h. At this time, the inter-vehicle relative distances are 11.58m and 0.7m respectively, indicating that the proposed DW-SB AEB control system has achieved emergency collision avoidance function in both two test conditions. As shown in Fig 17(d), the actual braking deceleration of the vehicle can track the expected braking deceleration well, indicating that the control effect of the lower layer RISMC expected braking deceleration tracking controller is great. Moreover, the expected braking deceleration curve of the vehicle is relatively smooth, and the maximum longitudinal jerks are 8.9m/s^3^ and 9.72m/s^3^ respectively, both of them are within the range of braking comfort.

In summary, the real vehicle experiment shows that the proposed DW-SB AEB control strategy can achieve emergency collision avoidance function safety in different test scenarios, while maintaining great braking comfort. It can effectively improve the safety and comfort of the vehicle and meet the application needs of commercial buses.

## 6. Conclusion

In response to the application requirements of safety and comfort in emergency braking of commercial buses, this paper studies the AEB control strategy considering comfort and draws the following conclusions:

(1) A DW-SB AEB Hierarchical control strategy architecture is proposed, in which an expected braking deceleration optimal decision controller based on PSO-LQR and comfort time compensation coefficient is designed in the upper layer, and an expected braking deceleration robust tracking controller based on feedforward and RISMC feedback control is designed in the lower layer. The HIL test results show that the proposed DW-SB control strategy can achieve emergency collision avoidance function for commercial buses. Under three typical working conditions of CCRs, CCRm and CCRb, compared to traditional DW-DB control strategy, it enhances the passenger comfort during the emergency braking while ensuring the vehicle's collision avoidance function safety.
(2) An AEB control strategy software architecture based on AUTOSAR is designed and completed the controller software development. The real vehicle test results showed that the AEB control strategy software can achieve the expected emergency collision avoidance function of the commercial buses, and maintain great riding comfort during the emergency braking, it possesses high practical value in engineering application.

While this study provides a valuable AEB control strategy that can improve vehicle safety and ride comfort during emergency, several limitations warrant acknowledgment. One is that the proposed AEB control strategy is only verified by simulation tests under uniform road conditions, and it is not fully verified under complex working conditions such as slippery low-adhesion roads and bisectional roads with changing adhesion coefficients. Another is that, the AEB control strategy is only verified by real vehicle tests in closed simple scenarios considering the actual test conditions and safety, and no real vehicle tests are carried out in complex real traffic scenarios. In the future, we plan to conduct experiments in professional venues, and verify the effectiveness of control strategy under complex driving conditions of commercial buses.
